# Conformational Landscape of the Di- and Tripeptide Permease A Transport Cycle

**DOI:** 10.1021/acs.jcim.5c00753

**Published:** 2025-06-09

**Authors:** Afshaan Kathrene Singh, Shruti Apurva, Khadiza J. Tazally, Chelsea K. D’Costa, Bala K. Prabhala, Shozeb Haider

**Affiliations:** † UCL School of Pharmacy, 371646 University College London, London WC1N 1AX, U.K.; ‡ Department of Physics, Chemistry and Pharmacy, University of Southern Denmark, Odense 5230, Denmark; § University of Tabuk (PFSCBR), Tabuk 47512, Saudi Arabia; ∥ UCL Centre for Advanced Research Computing, University College London, London WC1H 9RL, U.K.

## Abstract

Dipeptide and tripeptide permease A (DtpA) transporter is a bacterial homologue of the human PepT that is responsible for the uptake of di- and tripeptides from the small intestine and transports them across the cell membrane utilizing an inward-directed proton electrochemical gradient. Despite its importance, the structural dynamics governing the conformational transitions of DtpA remain poorly understood. In this study, we employed Adaptive Bandit enhanced sampling molecular dynamics simulations to investigate the five major conformational states of DtpA adopted during the transport cycle. We identified key metastable states and transitions underlying the transport cycle using Markov State Models (MSMs). Our findings reveal that intra- and interhelical interactions drive conformational changes by inducing bending and rotation of helices lining the pore, resulting in its opening and closure. This study explains the substrate transport mechanism in DtpA, enhancing our understanding of bacterial proton-dependent oligopeptide transporters (POTs) and opening new drug design and development opportunities.

## Graphical abstract

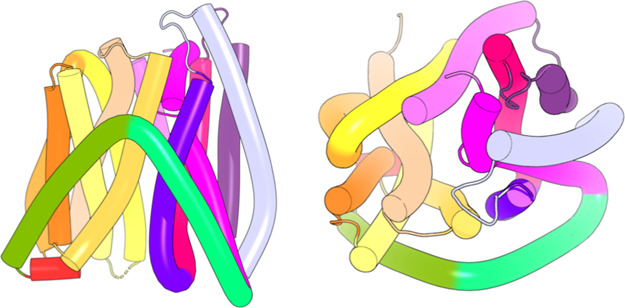

## Introduction

The transportation of small molecules across cellular membranes is a fundamental process that is necessary for sustaining life. Biological membranes, primarily composed of lipid bilayers, serve as barriers that separate the internal environment of the cell from its external surroundings. However, these membranes are selectively permeable as they allow only certain substances to pass through while restricting others.^1^ Small molecules such as ions, nutrients, and metabolic products often diffuse poorly across the lipid bilayer due to their size, polarity, or charge. To overcome this limitation, nature has evolved a highly sophisticated array of membrane transport proteins that function as gatekeepers, facilitating the controlled movement of specific molecules into or out of cells.^1^ Membrane transporters play a pivotal role in maintaining homeostasis by regulating the import of essential nutrients like glucose, amino acids, and ions as well as the export of waste products and toxins. These proteins act like dynamic gates, capable of opening and closing in a highly selective and regulated manner.^1^

The proton-dependent oligopeptide transporters (POTs) are transmembrane proteins that belong to a subfamily of the major facilitator superfamily (MFS), responsible for the uptake of dipeptides, tripeptides, and peptide-like compounds utilizing an inward-directed proton electrochemical gradient.^2^ POTs exhibit significant pharmaceutical value due to their conserved substrate-binding site, facilitating protein binding. Thus, they are associated with enhanced oral bioavailability of various drugs.^3,4^ Mammalian POTs are also called solute carrier 15 transporters (SLC15s).^5^ The two most important human POTs are PepT1 (SLC15A1) and PepT2 (SLC15A2) because of their role in the transport of di- and tripeptides and peptidomimetic drugs.^5^ They consist of 12 transmembrane helices and an extracellular domain.^6^ PepT1 is expressed in the small intestine and is involved in the intestinal uptake of peptides,^5^ while PepT2 is found in the kidneys and is responsible for renal reabsorption of the peptides.^7^

To understand the mechanism of peptide transport, an alternating access model was presented to describe how peptides translocate between Outward and Inward Open conformations.^8^ According to this hypothesis, the central cavity where the substrate binds is exposed to both the periplasmic and the cytoplasmic sides, allowing the substrate to be transported from one location to another. When a peptide enters the cavity, the gating helices undergo a significant conformational shift, causing the extracellular side to close and obstruct it. Protons moving from the extracellular side to the intracellular side enable the intracellular side to open and release peptides and protons inside the cell.^9^

The rocker-switch model was proposed as part of the alternating access model.^4^ This model was presented assuming that the two bundles comprising the central cavity are symmetrical around the binding site, although they are not. Furthermore, this model ignored the three intermediate conformations between the Outward and Inward open states, which resulted in it not being widely accepted.^10^

Another model explaining the mechanism of movement is the clamp and switch model.^11^ Based on the alternating access model, this model initially occludes the binding sites by bending the alpha helices at one end, resulting in a slight N- or C-bundle rotation. This process is known as clamping. Following that, the bundles rotate entirely, exposing the binding site on the other side of where the mechanism began. This is known as the switch mechanism. This model recognizes the conformations between the outward and the inward open states.^12^

Dipeptide and Tripeptide Permease A, or DtpA, is a transmembrane protein in Escherichia coli inner cell membrane and is involved in the bacterial transmembrane transport of di- and tripeptides.^13^ It belongs to the POT family and is homologous to the human protein PepT1.^13^ These proteins can identify and transport about 8000 tripeptides and all 400 potential dipeptides.^14^ Pharmacologically, they can locate and transfer a wide range of chemically complex therapeutic compounds, including protease inhibitors, β-lactam antibiotics, and antivirals, like valaciclovir. The oral bioavailability of these drugs can be increased by conjugating them with amino acids to form prodrugs, which are recognized and actively transported by peptide transporters like PepT1.^15^ This strategy leverages the transporter’s proton-coupled symport mechanism, enabling efficient intestinal uptake and subsequent intracellular breakdown to release the active drug, as shown by valaciclovir, which exhibits improved bioavailability compared to acyclovir.^15–17^

DtpA and PepT1 show similar ligand selectivity and most drugs targeting the human protein interact with DtpA as well, a unique characteristic not found in any other bacterial homologue of PepT1.^18^ In addition, differently charged peptides with the same side chains but different spatial orientations are known to bind to PepT1 with different affinities. Similar stereospecificity has been seen in DtpA interactions.^14^ DtpA can, therefore, be used to examine drugs that target human PepT1.^5^ E. coli expresses the gene YdgR, which encodes for DtpA protein.^13^ Since E. coli is a vital part of the human gut microbiome, the expressed protein may compete with drugs that target PepT1 if the proportion of the gene expressed in the bacteria is significant. This reinforces the significance of the bacterial protein analysis.^19^

DtpA has 14 helices, unlike the 12 helices found in mammalian POTs^1^ (Figure 1A). Helices 1–12 are divided into the N-terminal bundle (Helices 1–6) and the C-terminal bundle (Helices 7–12). The two extra helices in the bacterial proteins, HA (connected to the sixth helix) and HB (attached to the seventh helix), are the transmembrane alpha helices, which connect the N-terminal and the C-terminal. HA and HB form a hairpin structure and do not to contribute to the peptide transport mechanism in DtpA.^5^ These two helices also have the lowest sequence identity in the POTs family.^11^ The N- and C-terminal bundles form an inverted “V” shaped structure, opening either on the cytoplasmic or periplasmic side. Of the 12 helices, 3, 6, 9, and 12 play a structural role, while helices 1, 2, 4, 5, 7, 8, 10, and 11 are engaged in the mechanism of the peptide transportation.^20^ The substrate binding site of DtpA is well conserved.^5^ Five different conformations of the DtpA have been reported, namely, Outward Open, Outward Occluded, Occluded, Inward Occluded, and Inward Open (Figure 1B). These conformations show back-and-forth movement among each other. The peptide is transported from the outside of the cell to the inside by going from the Outward Open conformation to the Inward Open conformation. The peptide molecule is trapped within the pore in three intermediate conformations. DtpA functions as a proton-dependent peptide transporter, with its conformational dynamics intricately regulated by the proton gradient across the membrane.^20^ This mechanism involves the simultaneous uptake of a proton and a peptide substrate from the extracellular environment. Upon binding of a proton to the transporter, a conformational change occurs, leading to the occlusion of the pore. This state traps the substrate and the proton within the transporter, preventing backflow. Subsequently, when the proton dissociates from the transporter on the intracellular side, another conformational shift is triggered, which transitions the protein to an open state. This proton release facilitates the peptide substrate’s release into the cytoplasm. Thus, the proton gradient acts as a driving force, coupling proton movement to substrate transport through inducing structural changes in the protein.^21^ However, the conformational changes that occur during transitions are poorly understood due to the lack of structures.

**Figure 1:**
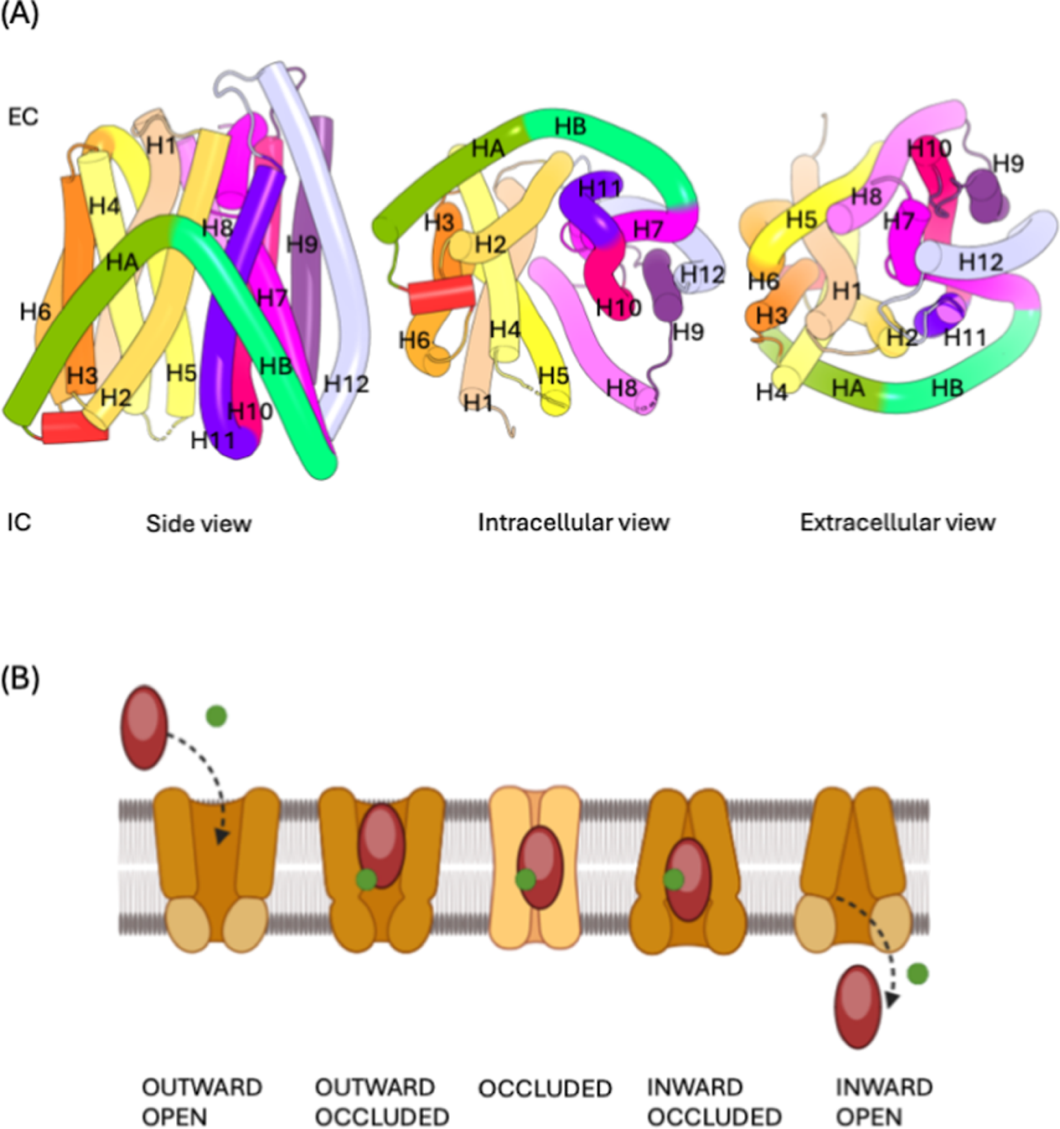
(A) Structure of the DtpA transporter of the Inward Open conformation (PDB 6GS4) illustrating side, intracellular (IC), and extracellular (EC) views. (B) Proton-dependent transport of peptide in DtpA through the five conformations—Outward Open, Outward Occluded, Occluded, Inward Occluded, and Inward Open. The brown oval represents the peptide and the green ball represents the proton.

The conformational states of DtpA remain largely uncharacterized, with existing structures limited only to the crystallographic inward-open state. Due to the lack of comprehensive structural data covering the full range of conformational states—from the Outward Open to the Inward Open states—in a single organism, we generated multiple homology models. We then examined the dynamics of the DtpA protein and the five distinct conformations that it adopts during peptide transport using enhanced sampling molecular dynamics simulations. Markov State Models (MSMs) were then employed to cluster the simulation trajectories, enabling us to identify any preferred conformations or pathways and to analyze transitions between states. The analysis enabled us to understand the structural transitions involved in the transport cycle.

## Methodology

### Generating Models of DtpA

Structural models were constructed for each of DtpA’s five conformational states without any ligands. The primary goal was to investigate the five major conformational states of DtpA. By excluding the ligand, the study isolates the protein’s intrinsic structural transitions driven by proton coupling and helical rearrangements, providing a baseline understanding of the mechanism without the influence of substrate binding. The experimentally resolved structures of DtpA were only available in the Inward Open conformation (PDB id 6GS4) but with missing residues.^5^ Therefore, the AlphaFold-predicted structure AF-P77304-F1 was utilized to complete this structure. Homology modeling was applied to model the other states. The target sequence of DtpA was obtained from UniProt (www.uniprot.org) with the identifier number P77304. The details of model construction of the other states have been provided in the Supporting Information (Table S1 and Figure S1). These models were analyzed and compared with each other to observe the residues that changed positions with the change in conformations. Here, we also carefully observed the residues of the helices lining the pore. This step was crucial for deciding a set of features to generate Markov State Models for each system. The terminal ends of the models were trimmed so that each model contained an equal number of residues from 19 to 484.

### Adaptive Bandit Enhanced Sampling Simulations

Before running the MD simulations, the models were protonated using the ProteinPrepare module implemented in PlayMolecule.^22^ A pH of 6.5 was employed rather than the physiological pH of 7.4 to mimic the acidic conditions of the small intestine and to account for the three protonated states of DtpA in the transport cycle. Residue E396 was protonated for the 3 Occluded conformations as its protonation was found to be involved in driving the conformational change from Outward to Inward Open in other POT species.^5^ The protonated models were embedded into a 1-palmitoyl-2-oleoyl-*sn*-glycero-3-phosphocholine (POPC) bilayer. The boundaries of the membrane were identified using the Orientations of Proteins in Membranes (OPM) database, where preoriented structures embedded into their respective membrane can be found.^23^ The HTMD package^24^ was used to embed the protein in the POPC bilayer. Following this, the system was solvated within a cubic TIP3P water box and built using the CHARMM 36m force field.^25^ Subsequently, the system was equilibrated for 50 ns in the *NPT* ensemble at 1 atmospheric pressure using a Berendsen barostat.^26^ The equilibration started with restraints on the protein backbone. The restraints were gradually decreased over 50 ns until no restraints were present.

For the production phase, the simulations were conducted in an isothermal–isobaric *NVT* ensemble using a Langevin thermostat with a damping constant of 0.1 ps^–1^ and a hydrogen mass repartitioning scheme, enabling 4 fs time steps. All bonds involving hydrogen atoms were constrained, consistent with simulations running with a larger time step. The initial velocities for each simulation were sampled from a Boltzmann distribution at 300 K. Adaptive Bandit enhanced sampling protocol employing multiple short simulations based on Markov State Models (MSMs) was used. The adaptive algorithm iteratively runs short parallel simulations, avoiding redundancy by discretizing the conformational space into an MSM and estimating the free energy from the stationary distribution of each state. Simulations are restarted from low-energy conformations. The MetricSelfDistance function evaluated native Cα contacts across residues to build the MSMs, with an exploration value of 0.01 and a goal-scoring function of 0.3. Each round included four 100 ns simulations accumulating over 20 μs, with trajectory frames saved every 0.1 ns. A total of 200 trajectories, each containing 3000 frames, were generated for each state. All simulations were performed using the ACEMD molecular dynamics engine.^24,27^

### Markov State Models

Markov State Models (MSMs) were built for each state using PyEMMA^28^ to study the conformational changes in DtpA. The sets of features that best described the slow dynamics of the system were selected. To approximate the slow dynamics of the system in a statistically efficient manner, a reduced-dimensional representation of the simulation data was crucial. Thus, all possible features were ranked based on the Variational Approach for Markov Processes (VAMP-2) scoring algorithm (Figure S2). In particular, a scalar score is obtained using VAMP to conveniently compare the ability of certain features to capture slow dynamics in a specific system.^28^ The best set of features was chosen, keeping in mind that the features should be constant throughout the five different systems. Finally, the features chosen were the position of the heavy atoms on the backbone, χ1 angles, and distances between 19 selected residues (8 on each side and 3 in the middle of each helix lining the pore). The selected residues were Q51 (Helix 1), E56 (Helix 2), A112 (Helix 4), K176 (Helix 5), N306 (Helix 7), E319 (Helix 8), V382 (Helix 10), and M441 (Helix 11) on the periplasmic side; A22 (Helix 1), K83 (Helix 2), C140 (Helix 4), G150 (Helix 5), G269 (Helix 7), N341 (Helix 8), Q409 (Helix 10), and L415 (Helix 11) on the cytoplasmic side; and Q41 (Helix 1), A45 (Helix 1), and P296 (Helix 7) in the center of the structure. Time-lagged independent component analysis (tICA) reduces the dimensions in the feature space, which usually contains many degrees of freedom, to a lower dimensional space that can be discretized with higher resolution and better statistical efficiency.^28^ tICA was used to reduce the dimensionality of the data. Clustering or discretization was performed by assigning each system an appropriate number of clusters. In this step, the reduced tICA coordinates were clustered into several discrete states using the k-means algorithm.

An important criterion for Markovian dynamics in the reduced space is that the implied timescales (ITS) are a constant function of lag time τ. From the ITS plot, the smallest possible MSM lag times that gave the best results were chosen. The parameters used to build the MSMs are listed in Table S2. The Bayesian MSMs were further validated using the Chapman–Kolmogorov (CK) test and the Implied TimeScale (ITS) plot (Figures S3–S7). Since the visualization of the full transition probability matrix *T* was difficult, it was coarse-grained into a smaller number of metastable states using the Perron Cluster–Cluster Analysis (PCCA+) method. The CK test and the ITS plots were used to validate the Markovianity of the models. An appropriate number of metastable states was decided by identifying separations in the ITS plot.

The study employed MSMs to analyze the conformational landscape of DtpA across five major states: Outward Open, Outward Occluded, Occluded, Inward Occluded, Occluded, and Inward Open. These conformations represent distinct macrostates in the transport cycle, each characterized by unique structural features and stability profiles. The MSMs identified multiple metastable states within each macrostate, with the number of metastable states varying (six for Outward Open, four for Outward Occluded, eight for Occluded, three for Inward Occluded, and nine for Inward Open). The primary objective was to characterize the most structurally distinct and functionally relevant states that describe the transport cycle. To achieve this, we analyzed all clustered conformations from every metastable state and made comparisons between each system. The chosen representative conformations correlated to the structural continuity of the next system in the transport cycle. For the Outward Open conformation, the fifth metastable state was chosen; for the Outward Occluded, the fourth metastable state; for the Occluded, the fourth metastable state; for the Inward Occluded, the third metastable state; and for the Inward Open, the ninth metastable state was chosen. From each of these metastable states, a single representative frame was chosen after all of the frames within that metastable state. The selection of a single frame facilitated structural visualization and analysis while focusing on the most stable conformation within each system. The postprocessing and the analysis of the results obtained from the MSMs were performed using different software and packages. The trajectories were visualized using PyMol (www.pymol.org) and VMD (http://www.ks.uiuc.edu/Research/vmd/). MoleOnline (https://mol.upol.cz/) helped to visualize and analyze the pore and the residues lining the pore and plot the translocation pore radius plots. MDCiao (https://proteinformatics.uni-leipzig.de/mdciao/) was used to analyze the interactions. Bendix (https://www.ks.uiuc.edu/Research/vmd/plugins/bendix/) was used to assess helical bending. MDLovoFit (https://m3g.github.io/mdlovofit/) was used to calculate the structural parameters like root mean-squared deviation and fluctuations.

## Results

The conformation of the dynamic pore characterizes the five distinct conformational states of DtpA. Each conformation represents a different structural state and is essential in understanding the peptide transport mechanism across the cellular membrane. The Outward Open conformation (Figure 2A) depicts the transporter in an open state toward the extracellular (periplasmic) side. In this state, the pore is wide open toward the periplasm. At the same time, the intracellular (cytoplasmic) side is shut, allowing the binding site to be exposed to the external environment, indicating that the pathway is open for the entry of the substrate and proton from the extracellular side. The pore is partially closed toward the extracellular side in the next conformation, representing the Outward Occluded conformation (Figure 2B). This state occurs after the peptide and proton are bound to the transporter. The structure of the pore in this conformation is such that the extracellular side closes, while the intracellular side remains shut. This closure ensures that the peptide and proton are enclosed within the transporter without escaping into the periplasmic environment.

**Figure 2:**
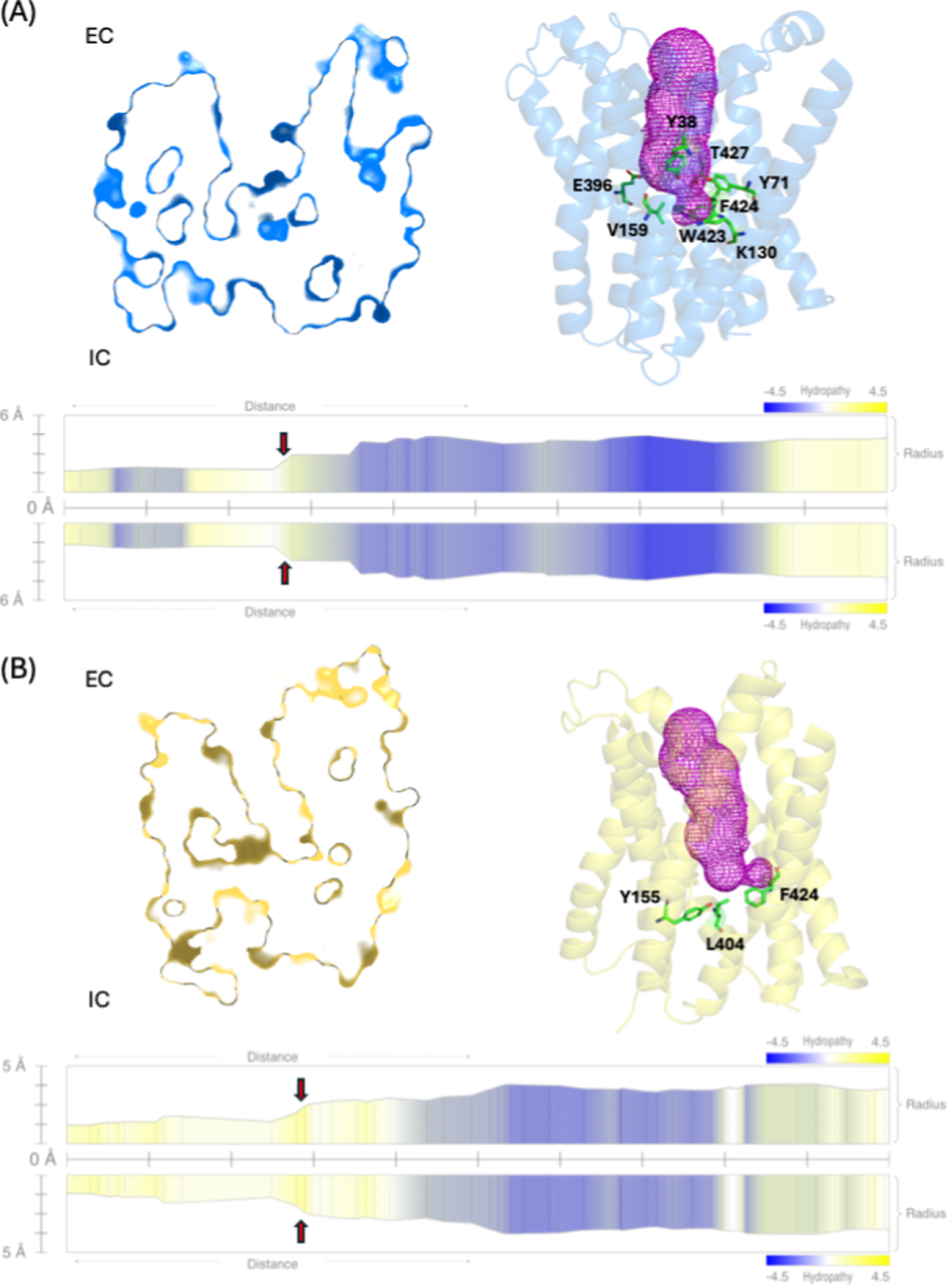
Representation of the Outward Open and the Outward Occluded conformations. (A) In the Outward Open conformation, the pore is wide open toward the extracellular (EC) environment, and the residues constricting the intracellular side are Y38, Y71, K130, V159, E396, W423, F424, and T427. (B) In the Outward Occluded conformation, the pore is partially open toward the EC side. The constricting residues on the intracellular (IC) side are Y155, L404, and F424 (red arrows on the translocation pore radius plots). The cross-sectional view (left) and the ribbon representations (right) are used to visualize the pore conformation. The constricting residues that line the pore (green sticks), and the internal surface of the pore (magenta mesh) are highlighted. The *Y*-axis in the translocation pore radius plots indicates pore radius (in Å), while the *X*-axis represents the translocation path from IC to EC (from left to right). The hydropathy profile overlays the radius plot, with blue regions indicating hydrophilic and yellow indicating hydrophobic regions. Both conformations exhibit a similar constriction zone, though differences in pore width and hydropathy distribution suggest variations in substrate accommodation and transport dynamics.

The Occluded conformation (Figure 3) is characterized by both the periplasmic and cytoplasmic ends being closed. It is an intermediate step in the peptide transportation mechanism of DtpA. It ensures that the substrate and the proton are trapped inside the transporter, isolated from both the extracellular and intracellular environments.

**Figure 3:**
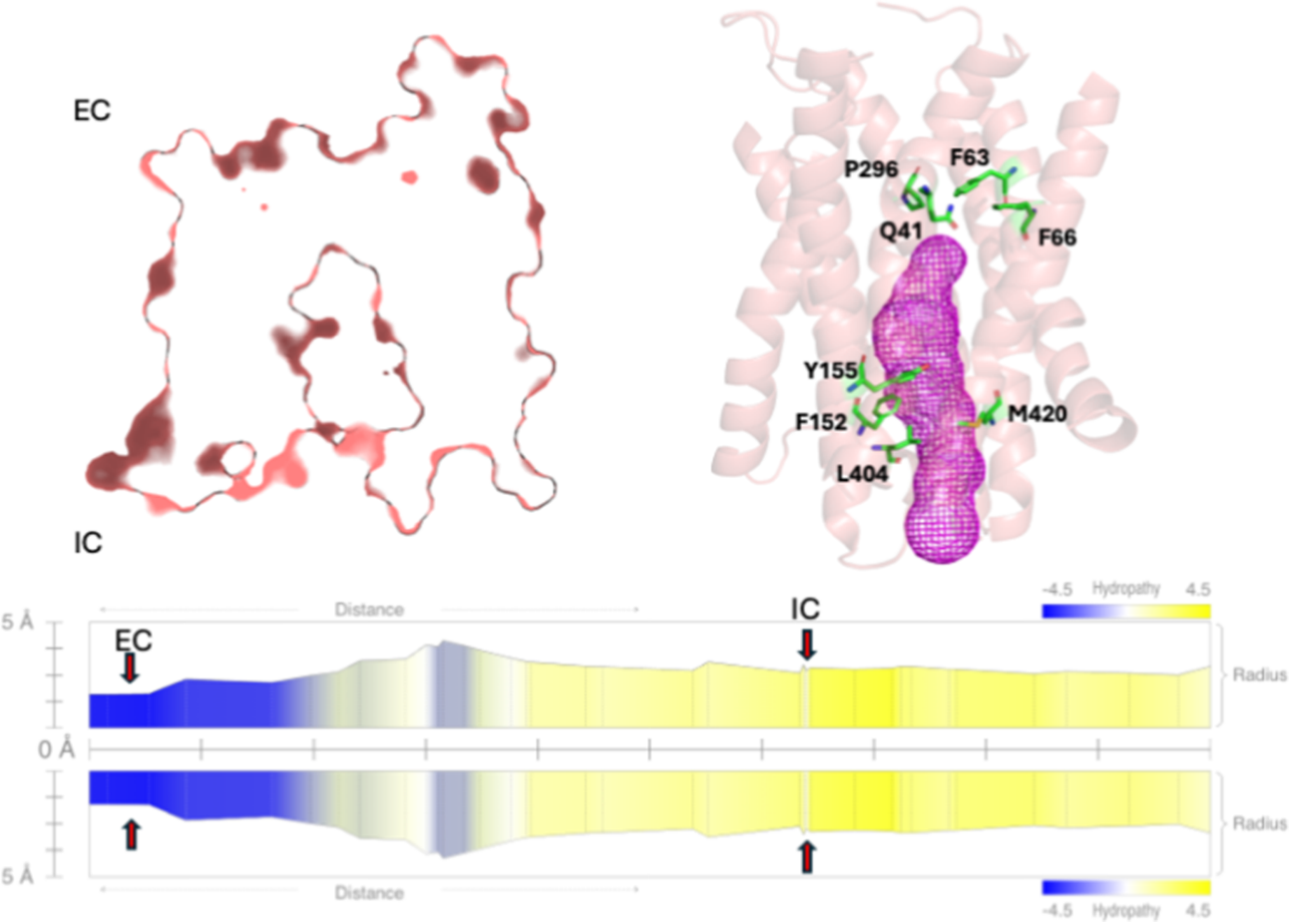
Representation of the Occluded conformation. In the Occluded conformation, the pore is constricted on both, extracellular and intracellular, sides by Q41, F63, F66, and P296 (EC) and F152, Y155, L404, and M420 (IC). The constricting residues that line the pore (green sticks), and the internal surface of the pore (magenta mesh) are highlighted. The constricting residues are depicted with arrows on the translocation pore radius plots. The *Y*-axis indicates the pore radius (in Å), while the *X*-axis represents the translocation path from EC to IC (from left to right). The hydropathy profile overlays the radius plot, with blue regions indicating hydrophilic and yellow indicating hydrophobic regions.

In the Inward Occluded conformation (Figure 4A), the transporter is partially open toward the inside of the cell. In this conformation, the pore transitions to an inward-facing state where the periplasmic end remains closed, but the cytoplasmic end begins to open. The Inward Open conformation (Figure 4B) leads to the final step of the transportation cycle, where the substrate and the proton are released into the cytoplasm because the pore in this conformation opens wide toward the intracellular space, leading to the peptide and proton exit from the transporter to the intracellular environment. This sequence of conformational changes in DtpA demonstrates the alternating-access model, ensuring that the transportation cycle is directional and regulated.

**Figure 4:**
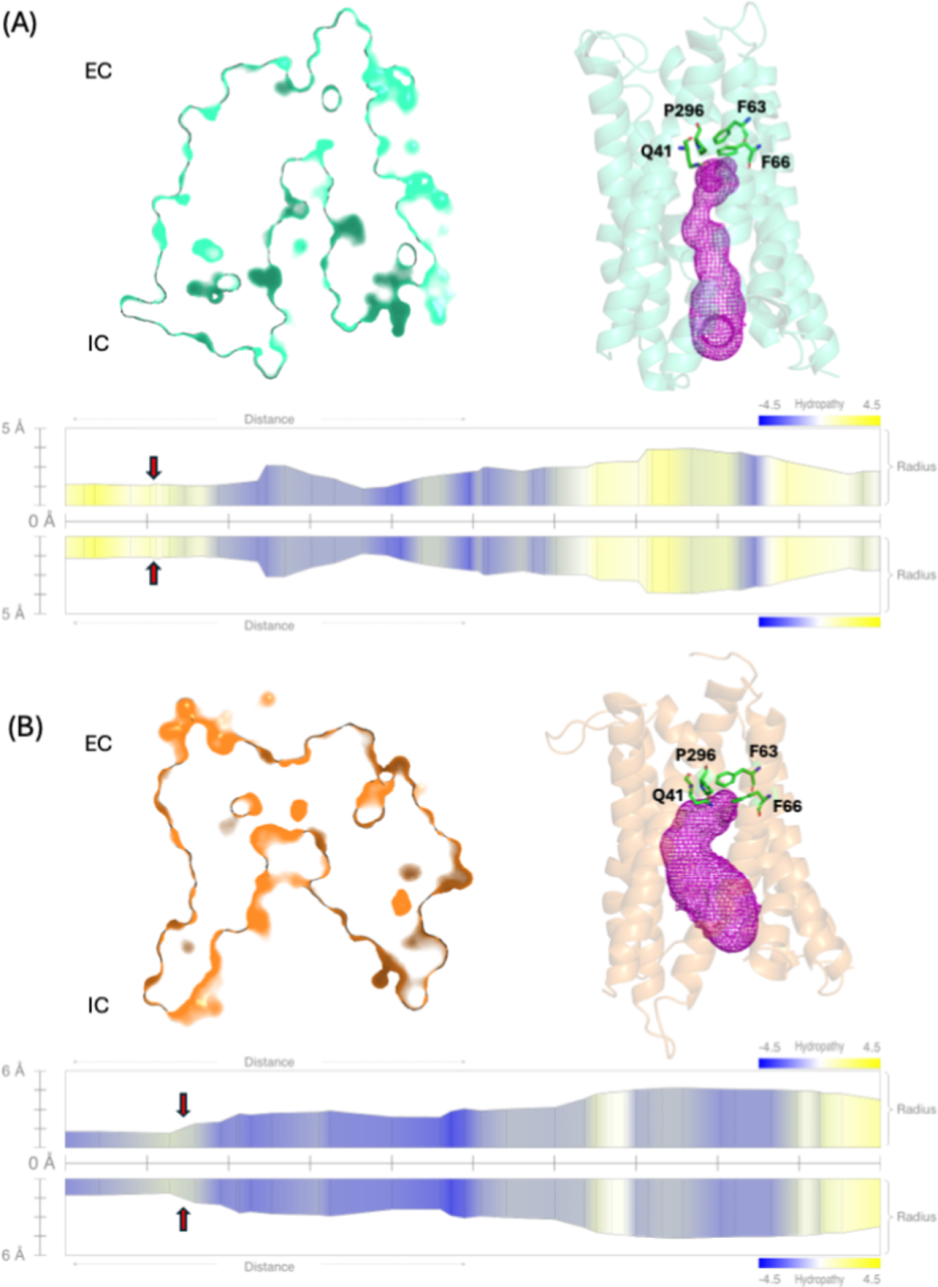
Representation of the Inward Occluded and Inward Open conformations. (A) In the Inward Occluded conformation, the pore is partially open toward the intracellular environment. The constricting residues on the extracellular side include Q41, F63, F66, and P296 (green sticks). (B) In the Inward Open conformation, the pore is wide open toward the intracellular side. In this state, the constricting residues are similar to those observed in the Inward Occluded state. The internal surface of the pore (magenta mesh) is highlighted. The *Y*-axis in the plot indicates pore radius (in Å), while the *X*-axis represents the translocation path from EC to IC (from left to right). The hydropathy profile overlays the radius plot, with blue regions indicating hydrophilic and yellow indicating hydrophobic regions. The constricting residues are depicted with arrows on the translocation pore radius plots. Both conformations exhibit a similar constriction zone, though differences in pore width and hydropathy distribution suggest variations in substrate accommodation and transport dynamics.

Salt bridges and hydrogen bonds are integral in stabilizing various conformational states of proteins, serving as key drivers of structural transitions.^29^ These noncovalent interactions between protein helices facilitate the fundamental conformational changes necessary for the protein’s function.^29,30^ The conformational dynamics of peptide transporters has previously been analyzed by calculating the frequency of salt bridge and hydrogen bond formation as well as contact patterns between residues in distinct states.^30^ We have extended a similar analysis to DtpA, which provides insight into how the conformational shifts occur and reveals specific residues that play essential roles in mediating these transitions. Among the residues critical for the formation of the pore on the extracellular side are E56, R305, P296, K50, I43, E317, N300, Q322, and Y389, which collectively play a vital role in regulating the opening and closure of the pore (Figure S8). These residues are positioned on helices 1, 2, 7, 8, and 10, emphasizing their importance in the conformational dynamics of the transporter. Together, these helices coordinate the conformational rearrangements essential for the gating mechanism on the extracellular side, demonstrating their role as a central structural element in transport regulation.

In the Outward Occluded, Occluded, Inward Occluded, and Inward Open states, two ion pair interactions between R305 (Helix 7)–E56 (Helix 2) and K50 (Helix 1)–E317 (Helix 8) support the closed conformation at the extracellular side (Figure S9). However, during the transition to the Outward Open conformation, R305 undergoes a conformational shift that disrupts its contact with E56, leading to its detachment from Helix 2. This repositioning induces an outward bend in Helix 7. Similarly, the K50–E317 ion pair is also broken. The bending effectively disrupts stabilizing interactions of the closed conformation, allowing the pore to open on the extracellular side to facilitate substrate uptake.

There are five key tyrosine residues that play an important role in the conformational dynamics of the pore. These include Y38 (Helix 1), Y71 (Helix 2), Y155 (Helix 5), Y156 (Helix 5), and Y292 (Helix 7) (Figure S10). In the Outward open state, where the cytoplasmic side is completely closed, several key hydrogen bonds are formed by tyrosine residues that play a crucial role in maintaining this conformation. Specifically, Y71 (Helix 2) and Y38 (Helix 1) form hydrogen bonds with R34 (Helix 1), while Y156 (Helix 5) forms a hydrogen bond with E396 (Helix 10), and Y292 (Helix 7) interacts with N325 (Helix 8) and E396 (Helix 10) (Figure S11). These hydrogen bonds prevent the tyrosine residues from facing the channel pore, effectively opening the pore to the periplasm. In the Outward Occluded conformation, when the hydrogen bonds between Y38 (Helix 1) and R34 (Helix 1) (Figure S12), as well as between Y292 (Helix 7) and N325 (Helix 8) (Figure S11), are disrupted, the tyrosine residues begin to orient toward the pore. As the protein shifts to the fully Occluded state, the hydrogen bond between Y292 (Helix 7) and N325 (Helix 8) is re-established, while the bond between Y71 and R34 is broken. However, the interaction between Y71 and K130 persists, helping to maintain the Occluded conformation. In this Occluded state, the tyrosine residues point into the pore (Figure S10). The transition from the Occluded state to the Inward Open state is driven by rotation of the surrounding helices. In both the Inward Occluded and Inward Open states, the tyrosine residues no longer form significant hydrogen bonds, allowing them to rotate away from the pore. This coordinated reorientation of the tyrosine side chains results in the opening of the cytoplasmic side, facilitating the substrate release (Figure S10).

The presence of two clusters of residues between helices results in the stabilization of the different conformational states. The first cluster involves D82 (Helix 2), R414 (Helix 11), R272 (Helix 7), and E263 (Helix B) (Figure S13). The ion pair interactions between R414 and E263 anchor Helix B to Helix 11, as evident in the crystallographic Inward Open state. The transition to an Outward Open conformation brings together Helices 2 and 7 toward Helices 11 and HB. This allows D82 and R272 to interact with R414 and E263. The coming together of the helices from both the N-terminal and C-terminal bundles facilitates the constriction of the pore at the cytoplasmic side. The second cluster of hydrogen bonds, positioned in the center of the structure, stabilizes the N-terminal bundle of the protein. This consists of residues E30, E33, and R34 from Helix 1 and N126 and K130 from Helix 4 (Figure S14). This network ensures the structural integrity of the N-terminal region during the Outward Open or Inward Open conformation.

A comprehensive overview of the structural details highlights key residue interactions stabilizing the five conformational states of DtpA (Figure 5). A dynamic interplay of salt bridges and hydrogen bonds drive structural transitions essential for peptide transport. In the fifth metastable state of the Outward Open conformation, the pore is wide open on the extracellular side, facilitating substrate and proton entry. Key residues K50 (Helix 1), E56 (Helix 2), P296 (Helix 7), R305 (Helix 7), Q322 (Helix 8), and Y389 (Helix 10) are oriented outward, creating a wide opening. The pore is constricted on the intracellular side with residues Y38 (Helix 1), Y71 (Helix 2), Y156 (Helix 5), and E396 (Helix 10) forming a tight network. Hydrogen bonds (Y71–R34, Y156–E396) stabilize this closed IC side, and residues like N160 (Helix 4) and S393 (Helix 11) are oriented inward, reinforcing the sealed end. In the fourth metastable state of the Outward Occluded conformation, the pore is partially open on the extracellular side, with E56, K50, and Q322 coming closer together. R305 (Helix 7) moves inward and forms an ion pair with E56 (Helix 2), stabilizing the partially occluded state. P296 remains central, but the overall pore radius is reduced compared to the Outward Open state, indicating the clamped phase of the transport cycle. The intracellular side remains closed, similar to the Outward Open state. Y38, Y71, and Y156 maintain their inward orientation, with additional constriction from R34 (Helix 1) and N325 (Helix 8). D82 (Helix 2), R414 (Helix 11), R272 (Helix 7), and E263 (Helix B) cluster contributes to further stability as the pore is inaccessible from the cytoplasmic side. In the fourth metastable state of the Occluded conformation, the pore is fully closed on the extracellular side, with Q322, E56, and P296 forming a tight seal. K50 (Helix 1) also forms an ion pair with E317 (Helix 8), and N300 (Helix 7) is positioned to further restrict access. This conformation traps the substrate and proton inside, isolating them from the extracellular environment. The intracellular side is also sealed with Y38, Y156, and R34 positioned inward, supported by E30 (Helix 1) and K130 (Helix 4). The D82-R414-E263 (Helix B) cluster reinforces the closed state, ensuring no escape to the cytoplasm. This state represents the fully occluded intermediate of the transport cycle. In the third metastable state of the Inward Occluded conformation, the extracellular side remains closed, similar to the Occluded state, with E56, R305, and P296 maintaining their spatial positions. Q322 and E317 continue to stabilize the sealed conformation, preventing the backflow of the substrate. The pore begins to open on the intracellular side, with Y38, Y156, and R34 starting to reorient away from the pore center. K130 and N325 show slight outward movement, and the interactions in the D82, R414, R272, and E263 cluster increase, allowing partial access to the cytoplasm. This state marks the transition to inward-facing conformations. In the ninth metastable state of the Inward Open conformation, the extracellular side remains closed, with E56, R305, P296, and Q322 maintaining the seal. The ion pairs (R305–E56) and hydrogen bonds (Y389–Q322–N300) ensure that the pore is inaccessible from the outside. The pore is wide open toward the cytoplasm, allowing substrate and proton release into the intracellular environment. Y38, Y156, and R34 rotate away from the pore, and N325 and K130 move outward, creating a large opening. Helix 11 (R414) is pulled toward Helix HB (E263), further opening the pore, with E396 (Helix 10) deprotonated, facilitating the final release step.

**Figure 5:**
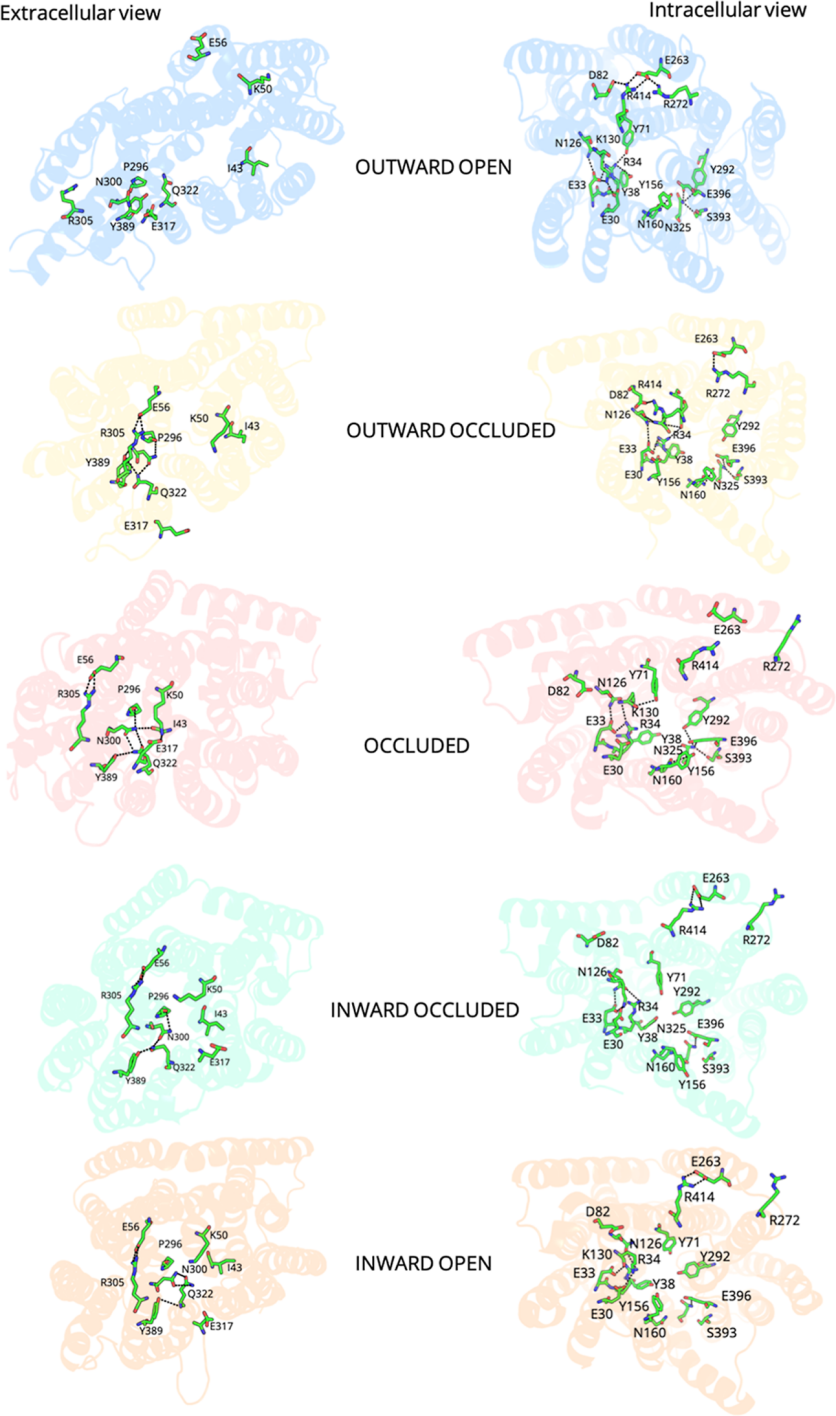
Interactions on the extracellular and intracellular sides in different conformational states.

## Discussion and Conclusions

The structure of DtpA demonstrates significant dynamic variability, resulting in five distinct conformations essential for peptide transport from the extracellular to the intracellular environment.^21^ These conformational states—Outward Open, Outward Occluded, Occluded, Inward Occluded, and Inward Open—exhibit structural dynamics, with the protein pore alternating between expansion and constriction. The continuous structural modulation is primarily driven by the bending and rotation of helices lining the protein pore. The helical movements are regulated by interactions between residues, which form and break noncovalent bonds. Such residue interactions lead to pore constriction and subsequent closure of the transport channel, both of which are vital for the protein’s transport mechanism.

Our results revealed that intra- and interhelical interactions drive conformational changes by inducing bending in helices lining the pore and rotating residues involved in gating the peptide channel. We applied the “clamp and switch” model to further elucidate the transport process and examine occluded states. Both inward- and outward-facing occluded conformations were observed, where the inward-facing conformations are marked by constriction at the periplasmic side and inward bending of helices near the cytoplasmic end, while the outward-facing structures show outward bending at the periplasmic side and constriction at the cytoplasmic side. According to the clamp and switch model, a slight rotation of the N- or C-terminal bundle occurs as the alpha helices bend, initially occluding the binding sites (the clamping stage).^31^ Subsequently, the bundles complete a full rotation, exposing the binding site on the opposite side—known as the switching mechanism.

To explore the mechanism driving peptide transport into the cell, we focused on the structural dynamics throughout the process, thereby uncovering the relationship between the protein’s functional role and the conformations it adopts. Members of the POT family exhibit several distinct conformations at various stages of the transport cycle, highlighting the importance of understanding each state to improve the targeted drug design. Targeting specific conformational states could lead to increased drug specificity and enhance bioavailability.

The Markov State Models revealed distinct, well-separated, and interconnected metastable states, exhibiting structural variation for each of the five conformations. This diversity in states reveals the broad range of structural dynamics within and between the conformations, which further highlights the mechanism of the transport of the peptide through the DtpA protein. We observed that helices 1, 2, 4, 5, 7, 8, 10, and 11 are involved in the pore dynamics. Bending and rotating these helices that line the protein’s pore drives this continuous structural change. These movements are influenced by interactions between specific residues (E56, R305, P296, K50, I43, E317, N300, Q322, Y389, Y38, Y71, Y155, Y156, Y292, R34, N160, N325, K130, D82, R414, R272, E263, E30, E33, N126, E396, S393), involved in the interhelical bond formation within these helices, which include the formation and breaking of bonds which cause residue rotation. These interactions eventually cause the pore to constrict and the transport channel to close, both of which play important roles in the protein’s transportation mechanism.

On the outward side of the protein, it is Helix 7, which drives the alteration in the conformations. When the extracellular side of the pore is closed, Helix 7 interacts with Helix 2 and Helix 8 with Helix 1 via R305 of Helix 7 and E56 of Helix 2, E317 of Helix 8 and K50 of Helix 1, and Y389 and Q382 of Helix 8, bringing the two terminals enclosing the pore closer and leading to the constriction of the pore. During the Outward Open conformation, there is a significant rotation seen in E56 of Helix 2 and K50 of Helix 1, resulting in Helix 7 losing contact with Helix 2 and Helix 8 losing contact with Helix 1, which stabilizes the opening of the pore on the extracellular side.

There are five tyrosine residues located on Y38 (Helix 1), Y71 (Helix 2), Y155 (Helix 5), Y156 (Helix 5), and Y292 (Helix 7). The positions of these tyrosine residues are stabilized by interactions with neighboring residues (R34, K130, N160, and N325). When they are not engaged in interactions, they reorient away from the pore, opening the channel on the extracellular and intracellular sides. In addition to these residues, a network of hydrogen bonds among residues in helices 1, 2, 4, 8, 10, and 11 (which are E33, R34, D82, N126, E263, R272, S393, E396 and R414) helps maintain the structural integrity across the five conformational states adopted by the protein.

In this study, we have used five different conformations as starting structures (PDB ids 7PMX for Outward Open, 7PMW for Outward Occluded, 4D2D for Occluded, 5OXL for Inward Occluded, and AF-P77304-F1 for Inward Open) for enhanced sampling simulations. Most of the bacterial POT structures are available as inward open or partially occluded. While we are able to account for the mean first passage times between various structural rearrangements within the same system, our study cannot estimate transition times between different systems. Nevertheless, to comprehensively understand the structural transition mechanisms of DtpA, it is necessary to analyze all five conformational states collectively. The cumulative conformational sampling of over 100 μs that is presented here provides us with an understanding of the dynamic motions and highlights how specific interactions influence the transport process, potentially providing unique insights that could apply to other human peptide transporters.

The results presented here are in excellent agreement with the experimental data. Previous observations in YdgR established that mutation of E396 abolished the transport of reporter substrate Beta-Ala-Lys­(AMCA).^19^ Similarly, conserved tyrosine residues and the ExxERFxYY motif are essential for function^32^ as the uptake of the beta-Ala-Lys­(AMCA) was either completely inhibited or reduced when these residues were mutated. However, mutational studies barely emphasize the importance of the residues while giving no information about their impact on the conformational changes. Despite advances in structural and functional studies, a unified mechanism explaining all POT conformations remains elusive.

Bacterial POTs like DtpA presently lack structures in complex with reporter substrate Beta-Ala-Lys­(AMCA) and antibiotics. Moreover, even the slightest change in the structure of Beta-Ala-Lys­(AMCA) was not tolerated by YdgR.^33^ Gaining more knowledge on the mechanism and dynamics of the active site can facilitate the design of drugs that effectively target specific conformational states of membrane transport proteins. The present study could be regarded as the first steps toward the development of a unifying mechanism of DtpA.

## Supplementary Material



## Data Availability

All files to run the simulations (input, psf, pdb, parameter), analysis scripts, and metastable state models can be downloaded using the DOI 10.5281/zenodo.15133526.

## Notes

Methods, list of POT structures; MSM parameters; validation of models; VAMP scores; MSM statistics; interactions in the Occluded conformation; tyrosine in Occluded and Inward Open states; tyrosines in the Outward Open state; interactions in Outward Open and Outward Occluded states; interactions in cluster 1; and interactions in cluster 2 (PDF)
