# Supplementary material for: Conformational Landscape of the Di- and Tripeptide Permease A Transport Cycle
Source: J Chem Inf Model. 2025 Jun 9;65(12):6198–208. doi: 10.1021/acs.jcim.5c00753 (PMC12199297; doi:10.1021/acs.jcim.5c00753)
Supplement: Supplementary file 1 [file ci5c00753_si_001.pdf]

Supporting Information

Conformational landscape of the Di- and Tripeptide Permease A (DtpA) transport cycle

Afshaan Kathrene Singh,[1] Shruti Apurva,[1] Khadiza J. Tazally,[2] Chelsea K D'Costa,[1] Bala K. Prabhala,[2] Shozeb Haider [1,3,4]

[1] UCL School of Pharmacy, University College London, London WC1N 1AX, U.K

[2] Department of Physics, Chemistry and Pharmacy, University of Southern Denmark, Odense, Denmark

[3] University of Tabuk (PFSCBR), Tabuk, Saudi Arabia

[4] UCL Centre for Advanced Research Computing, University College London, WC1H 9RL, U.K.

Corresponding author and email address
shozeb.haider@ucl.ac.uk

METHODS

Homology modelling

To better understand the conformational changes in DtpA, five conformations representing the transport cycle were required. The experimentally resolved structures of DtpA were only available in the inward open conformation (PDB id 6GS4) but were not completely resolved. Therefore, the AlphaFold predicted structure AF-P77304-F1 (Jumper et al., 2021) was utilised for this study as 93.4% of residues were modelled with confidence pLDDT >70

Homology modelling was applied to represent the other states. The target sequence of DtpA was obtained from UniProt (The UniProt Consortium, 2022) with the identifier number P77304 (500 residues) The next step was choosing templates homologous to DtpA but also represented each of the four conformations needed to model.

InterPro (Paysan-Lafosse et al., 2023) was used to find every structure from the POT family as every other family is the MFS superfamily is distinct. In total, 60 structures were identified. Each of them was sorted into each of the five states. States were determined based on the respective literature and inspection of the channel for each of the templates. Widening of the inward side of the pore and the constriction of the outward side of the pore as we go from the outward open to inward open states, were the hallmarks used to categorize the templates. This was done using MOLE software (Pravda et al., 2018).

For each potential template, sequence alignment was performed using a pairwise sequence alignment tool EMBOSS Needle (Rice et al., 2000). It was found that the percentage identity between the DtpA sequence and each template ranged from 19 to 28%. For a good model, often, sequence identity of over 40% is recommended for assurance that they have a similar fold. POTs are known to have low sequence identity among each other, but they do have a conserved architecture of the 12 or 14 transmembrane helices (Guettou et al., 2013). Therefore, the TM-align (Zhang and Skolnick, 2005) score was determined between the chosen inward open structure and each of the templates. TM-align is a structural alignment tool which optimally superimposes the two structures and returns a structural similarity score. It was decided to align each template to the selected AlphaFold inward open structure as the models will have different conformations based on this structure. Finally, the amount of unmodelled sequences and the resolution of each template was determined from the structural information on the Protein Data Bank (Berman et al., 2000). Based on considering each of these criteria, appropriate templates were chosen.

To model each of the conformations, MODELLER 10.4 (Webb and Sali et al., 2016) was used. The inputs were the target sequence of DtpA and the template PDB structure. 100 models were generated per conformation. The model with the lowest Discrete Optimised Protein Energy (DOPE) score was chosen.

The next step involved comparing the helix lengths of each model to those of the inward open structure. It was also checked whether the residues were aligned similarly to the inward open structure. This is a crucial step as a shift in even a single amino acid residue can affect the formation of a salt bridge or change the structure of the binding site which ultimately affects the dynamics. To fix this, a few output files with the lowest DOPE score of the first modelling run were used in addition to the inward open structure to run a multiple-sequence alignment. The models chosen here were those with the closest RMSD score to the template while also having the sequences aligned closely to the inward open structure. If some residues formed coils instead of expected helices, the restraint function was used to force helical formation wherever necessary based on inputted ranges of sequences.

The terminal residues of each of the 5 structures were trimmed so each model contained residues from residue 19 to 484. This was done as the trimmed out residues in the inward open AF-P77304-F1 structure was modelled with low to very low confidence. Additionally, this was done in preparation for molecular dynamics simulations to allow for the protein to be embedded within the membrane boundaries.

Upon visualisation of each of the selected templates, it was found that helices HA and HB of the inward open conformation were distinct in comparison to the templates, therefore, it was decided that the HA and HB modelled thus far were to be deleted and the structure of HA, HB and the coil structure between helix 6 and HA would be extracted from the inward open structure to be modelled onto each respective model that was made. Following the initial steps for modelling. It was double checked to make sure the sequences of extracted HA and HB from inward open structure aligned with the empty row of residues in each model alignment file before running the programme.

After assessing the templates, 5OXL was chosen to model inward occluded state which had an RMSD of 1.08 with the template. For the occluded conformation, multiple template modelling was performed with 4D2D which resulted with an RMSD of 1.07 and 1.46 with each respective template. As there was only one template for the outward occluded state, a model was made from the human PepT1 structure 7PMW which had an RMSD of 2.01. Finally, for the outward open conformation, the human 7PMX was chosen as the template which after modelling showed an RMSD of 1.01.

A distinctive aspect of our modelling approach involved the integration of HA and HB, from the inward open structure into each model. This inclusion extended to the coil connected Helix 6 and HA regions. This comprehensive approach, while enriching our models, could account for the RMSDs exceeding the 1 Å threshold in comparisons between the models and templates. The first step of validation was to check whether the helices of the model align with those in the inward open structure as mentioned above. It was expected that there will be structural difference such as bending of the helices, but this check was to make sure the sequences were placed in similar positions to the inward open structure. Following this, Ramachandran plots were obtained for each model to further validate them.

Table S1. POTs structures representing the potential templates for the inward occluded, occluded, outward occluded and outward open conformations. The table describes different criteria considered while selecting the appropriate templates. The chosen templates are highlighted in red

**PDB id**	**Experiment Type**	**Resolution (Å)**	**Protein Length**	**Incomplete residues**	**% Sequence Identity**	**TM-align score**
Inward occluded						
7PMT	Cryo-EM	3.80	729	0	22	0.54
2XUT	X-ray	3.62	516	31	21	0.73
5OXL	X-ray	2.66	483	6	23	0.78
5OXK	X-ray	2.38	483	27	23	0.78
Occluded						
4D2C	X-ray	2.47	483	35	24	0.77
4D2D	X-ray	2.52	483	8	23	0.83
5D59	X-ray	2.40	483	33	23	0.80
5OXM	X-ray	2.29	483	25	23	0.79
5OXP	X-ray	2.37	483	16	23	0.81
Outward occluded						
7PMW	Cryo-EM	4.10	708	17	24	0.68
Outward open						
7PMX	Cryo-EM	3.50	708	13	23	0.68
7PN1	Cryo-EM	3.90	708	13	22	0.44

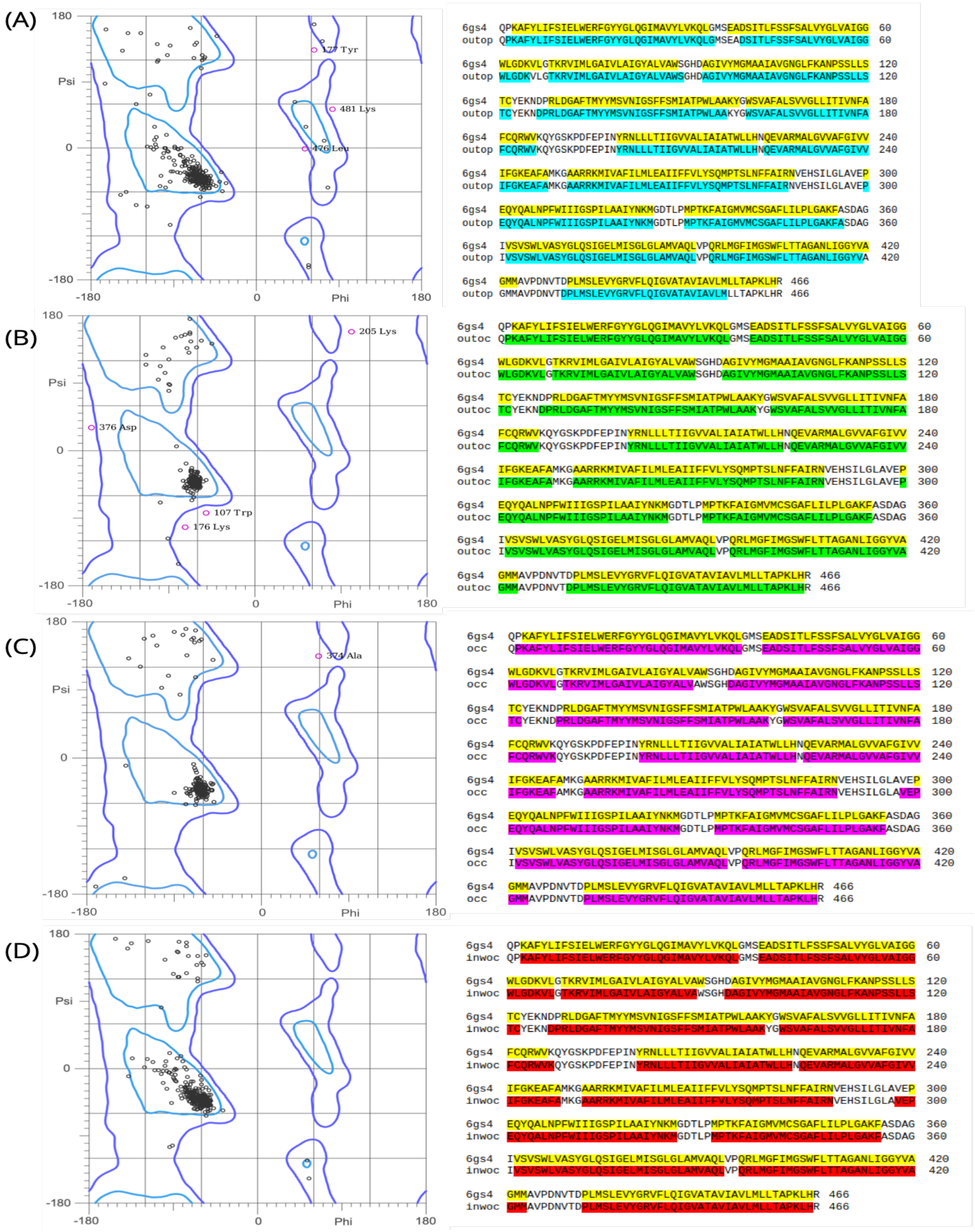

Supporting Fig. S1: Validation of the homology models. The Ramachandran plots (left) describe the torsional angles of each residue contained in each model. The alignments (right) illustrate the similarity between the helix lengths and positions between the inward open structure (PDB id 6GS4) and each model. Highlighted yellow is the reference structure (PDB id 6GS4) and their helix lengths. The other colours represent the helix lengths of the models.

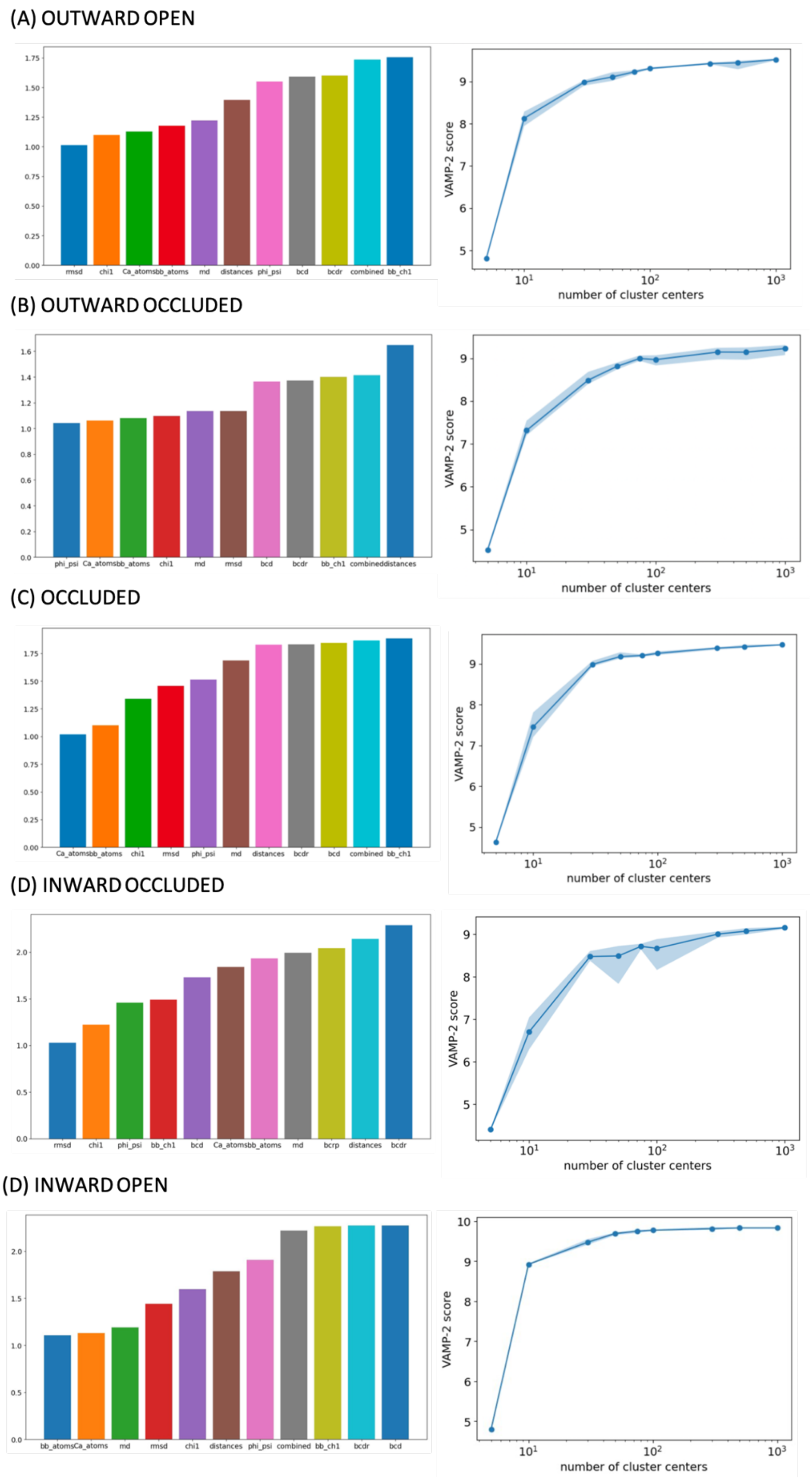

Supporting Fig. S2: VAMP Scores to evaluate the models

Feature 1: phi_psi represents all backbone dihedral angles

Feature 2: bb_atoms represents position of all backbone atoms

Feature 3: Cα_atoms represents position of all Cα atoms

Feature 4: rmsd represents Roots mean square Deviation

Feature 5: chi1 represents torsion of χ1angle
Feature 6: bb_ch1 represents torsion of backbone and χ1angles
Feature 7: distances represents Distances
Feature 8: bcd represents backbone, χ1 and distances torsions
Feature 9: bcdr represents backbone, χ1, distances and rmsd
Feature 10: md represents mdist is minimum distances between selected residues
Feature 11: combined represents all 10 features listed above combined

Table S2: Summary of the Markov State Model parameters

System	Conformation	TICA lag time (ns)	Clusters	MSM lag time (ns)	No. of States	No. of Trajectories (x 100ns)	Representative Metastable States
1	Outward Open	80	1000	2	6	212	5
2	Outward Occluded	80	1000	6	4	212	4
3	Occluded	70	100	4	8	211	4
4	Inward Occluded	50	100	2	3	228	3
5	Inward Open	4	500	1	9	232	9

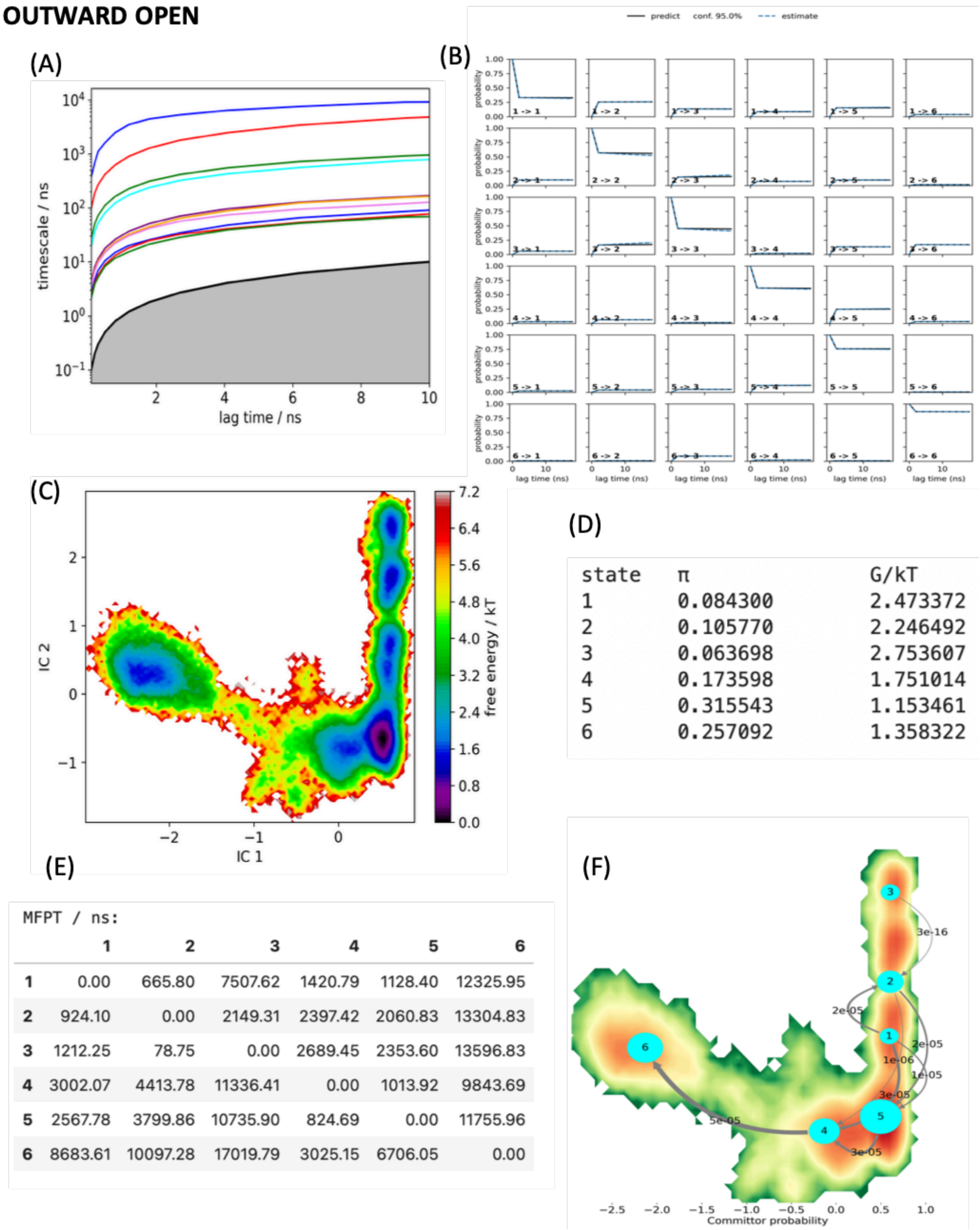

state	π	G/kT
1	0.084300	2.473372
2	0.105770	2.246492
3	0.063698	2.753607
4	0.173598	1.751014
5	0.315543	1.153461
6	0.257092	1.358322

MFPT / ns:

	1	2	3	4	5	6
1	0.00	665.80	7507.62	1420.79	1128.40	12325.95
2	924.10	0.00	2149.31	2397.42	2060.83	13304.83
3	1212.25	78.75	0.00	2689.45	2353.60	13596.83
4	3002.07	4413.78	11336.41	0.00	1013.92	9843.69
5	2567.78	3799.86	10735.90	824.69	0.00	11755.96
6	8683.61	10097.28	17019.79	3025.15	6706.05	0.00

Supporting Fig. S3: MSM of the Outward Open conformation. (A) The Implied Time Scale plot; (B) The CK plot; (C) The free energy surface plot (D) The state population and energies; (E) The mean first passage times and (F) The flux plot.

OUTWARD OCCLUDED

(A)

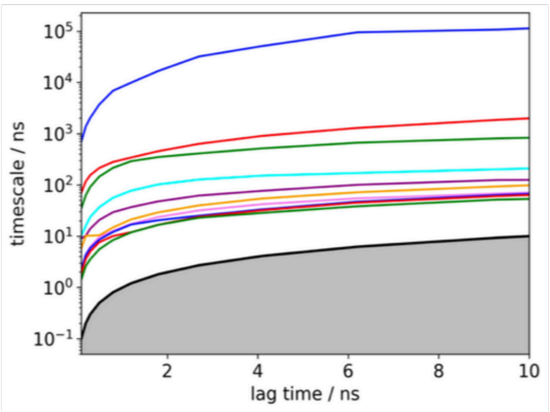

(B)

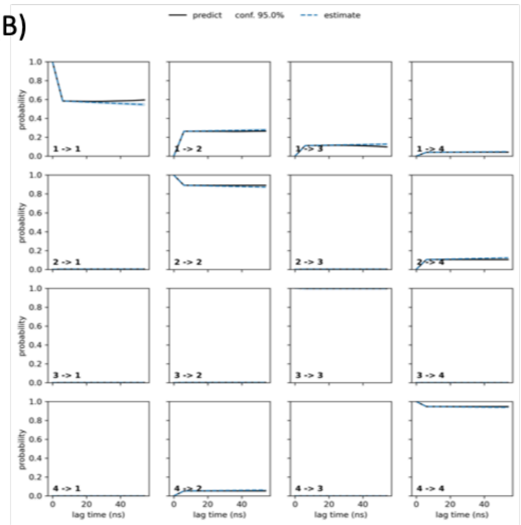

(C)

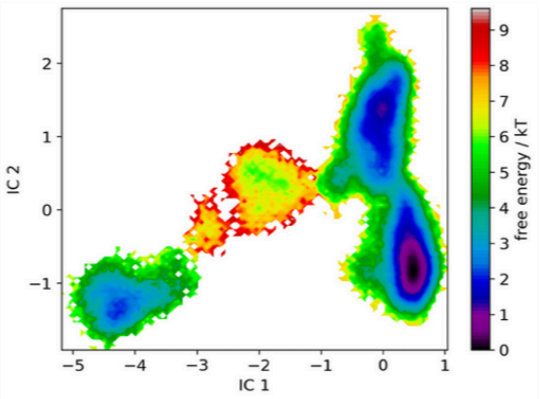

(D)

state	π	G/kT
1	0.001866	6.284028
2	0.128324	2.053197
3	0.307129	1.180488
4	0.562681	0.575042

(E)

MFPT / ns:

	1	2	3	4
**1**	0.00	43015.94	2876.62	25347.90
**2**	100232.29	0.00	32179.95	57109.88
**3**	218328.27	192618.41	0.00	2247.44
**4**	222297.09	196616.38	3063.11	0.00

(F)

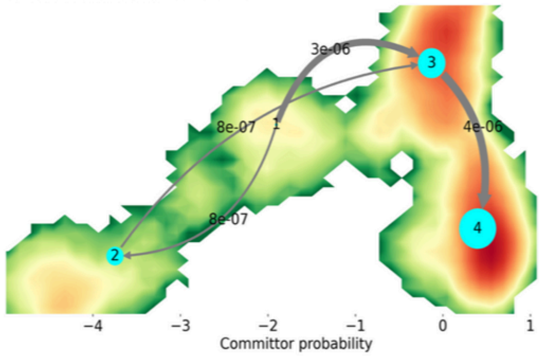

Supporting Fig. S4: MSM of the Outward Occluded conformation. (A) The Implied Time Scale plot; (B) The CK plot; (C) The free energy surface plot (D) The state population and energies; (E) The mean first passage times and (F) The flux plot.

OCCLUDED

(A)

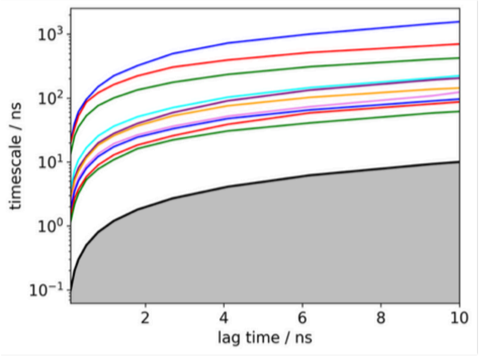

(B)

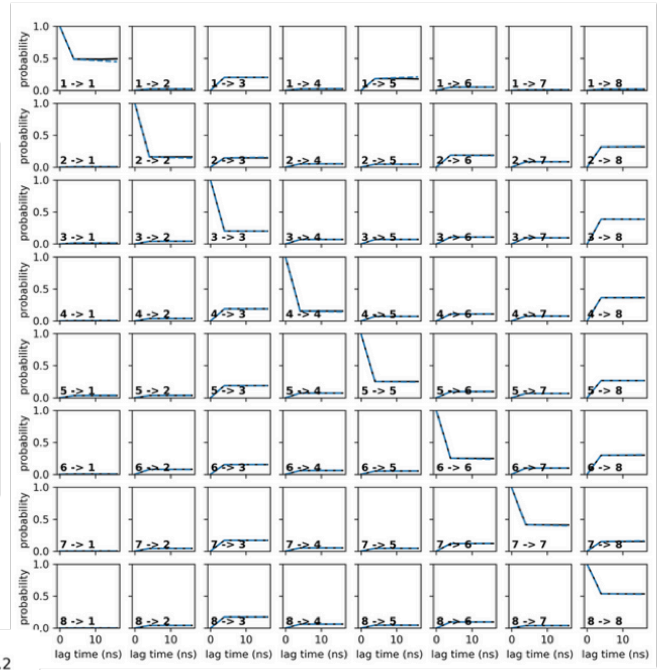

(C)

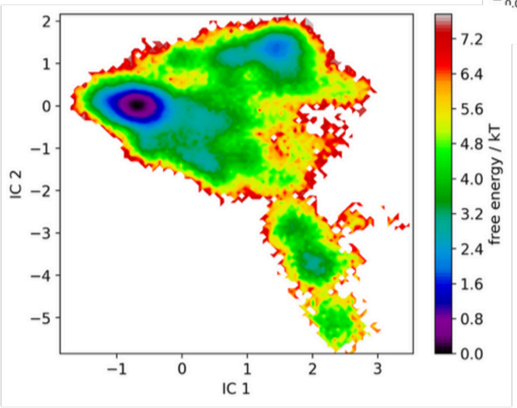

(D)

state	π	G/kT
1	0.010728	4.534902
2	0.012709	4.365411
3	0.021210	3.853299
4	0.020709	3.877209
5	0.055656	2.888564
6	0.100284	2.299751
7	0.176491	1.734485
8	0.602213	0.507143

(E)

MFPT / ns:

	1	2	3	4	5	6	7	8
**1**	0.00	10363.69	3727.13	19241.65	10920.80	872.66	1272.31	456.18
**2**	148.92	0.00	3845.61	19364.34	11043.49	997.64	1394.34	575.56
**3**	12962.90	27146.30	0.00	18589.33	10268.35	577.79	1022.84	118.90
**4**	14183.01	28357.45	4306.69	0.00	138.99	770.44	1708.93	1357.85
**5**	14019.28	28193.72	4142.94	6938.09	0.00	606.67	1545.22	1194.12
**6**	12983.03	27152.17	3352.48	17689.54	9368.65	0.00	692.65	238.23
**7**	13128.99	27305.69	3623.00	18596.52	10275.82	478.40	0.00	306.58
**8**	12861.34	27044.20	3292.11	18908.82	10587.96	642.74	945.86	0.00

(F)

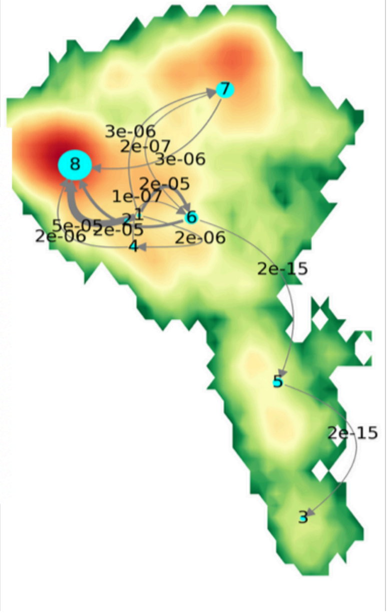

Supporting Fig. S5: MSM of the Occluded conformation. (A) The Implied Time Scale plot; (B) The CK plot; (C) The free energy surface plot (D) The state population and energies; (E) The mean first passage times and (F) The flux plot.

INWARD OCCLUDED

(A)

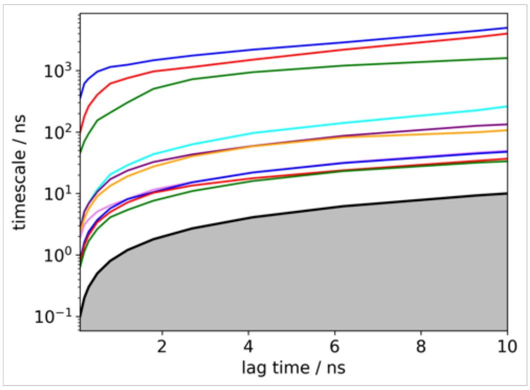

(B)

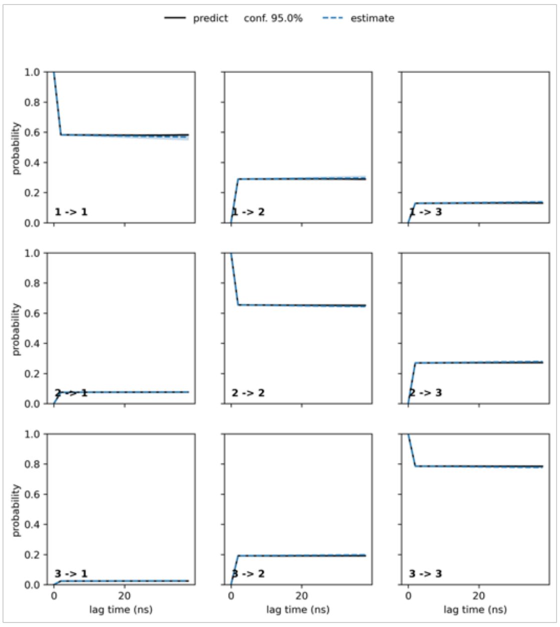

(C)

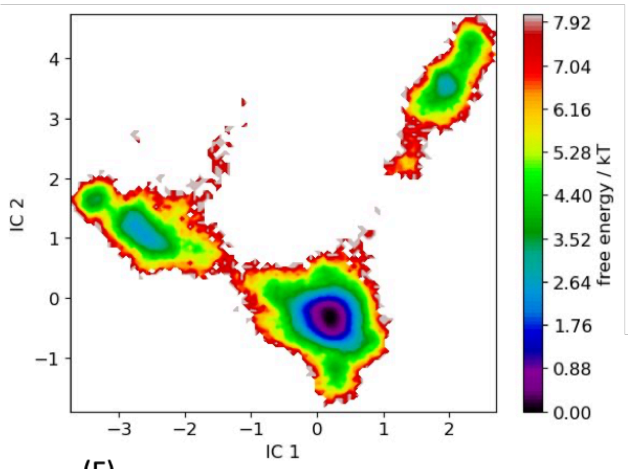

(D)

state	π	G/kT
1	0.068088	2.686948
2	0.570262	0.561660
3	0.361650	1.017078

(E)

MFPT / ns:

	1	2	3
1	0.00	1319.28	4098.53
2	12461.90	0.00	2779.14
3	14048.79	1511.71	0.00

(F)

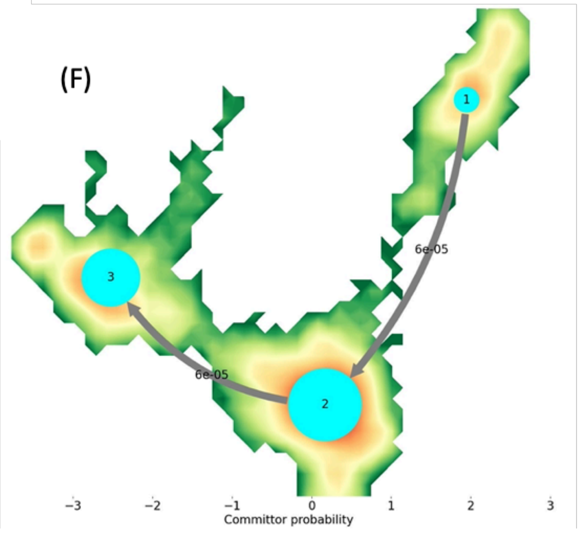

Supporting Fig. S6: MSM of the Inward Occluded conformation. (A) The Implied Time Scale plot; (B) The CK plot; (C) The free energy surface plot (D) The state population and energies; (E) The mean first passage times and (F) The flux plot.

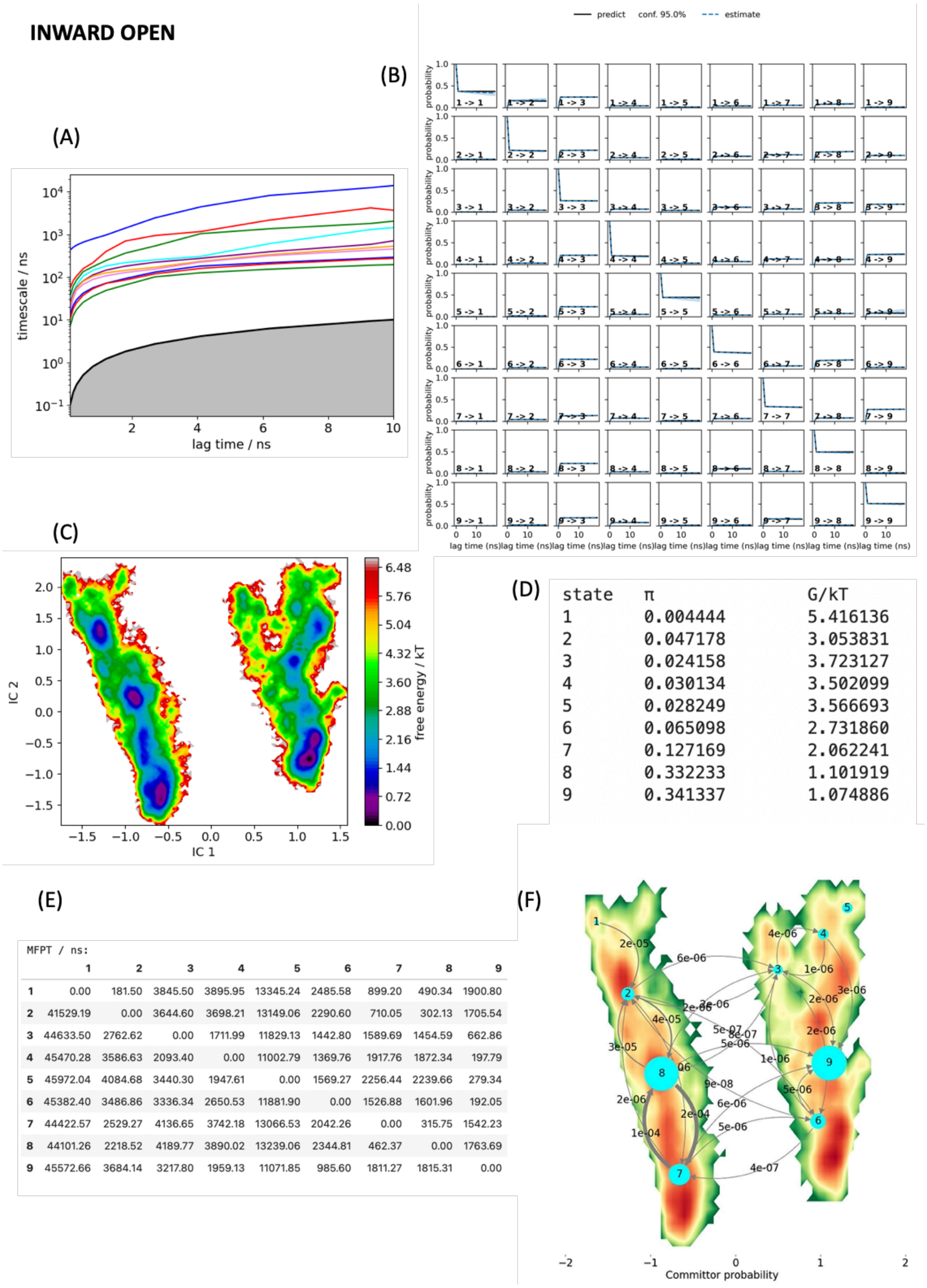

(D)

state	π	G/kT
1	0.004444	5.416136
2	0.047178	3.053831
3	0.024158	3.723127
4	0.030134	3.502099
5	0.028249	3.566693
6	0.065098	2.731860
7	0.127169	2.062241
8	0.332233	1.101919
9	0.341337	1.074886

(E)

MFPT / ns:

	1	2	3	4	5	6	7	8	9
1	0.00	181.50	3845.50	3895.95	13345.24	2485.58	899.20	490.34	1900.80
2	41529.19	0.00	3644.60	3698.21	13149.06	2290.60	710.05	302.13	1705.54
3	44633.50	2762.62	0.00	1711.99	11829.13	1442.80	1589.69	1454.59	662.86
4	45470.28	3586.63	2093.40	0.00	11002.79	1369.76	1917.76	1872.34	197.79
5	45972.04	4084.68	3440.30	1947.61	0.00	1569.27	2256.44	2239.66	279.34
6	45382.40	3486.86	3336.34	2650.53	11881.90	0.00	1526.88	1601.96	192.05
7	44422.57	2529.27	4136.65	3742.18	13066.53	2042.26	0.00	315.75	1542.23
8	44101.26	2218.52	4189.77	3890.02	13239.06	2344.81	462.37	0.00	1763.69
9	45572.66	3684.14	3217.80	1959.13	11071.85	985.60	1811.27	1815.31	0.00

Supporting Fig. S7: MSM of the Inward Open conformation. (A) The Implied Time Scale plot; (B) The CK plot; (C) The free energy surface plot (D) The state population and energies; (E) The mean first passage times and (F) The flux plot.

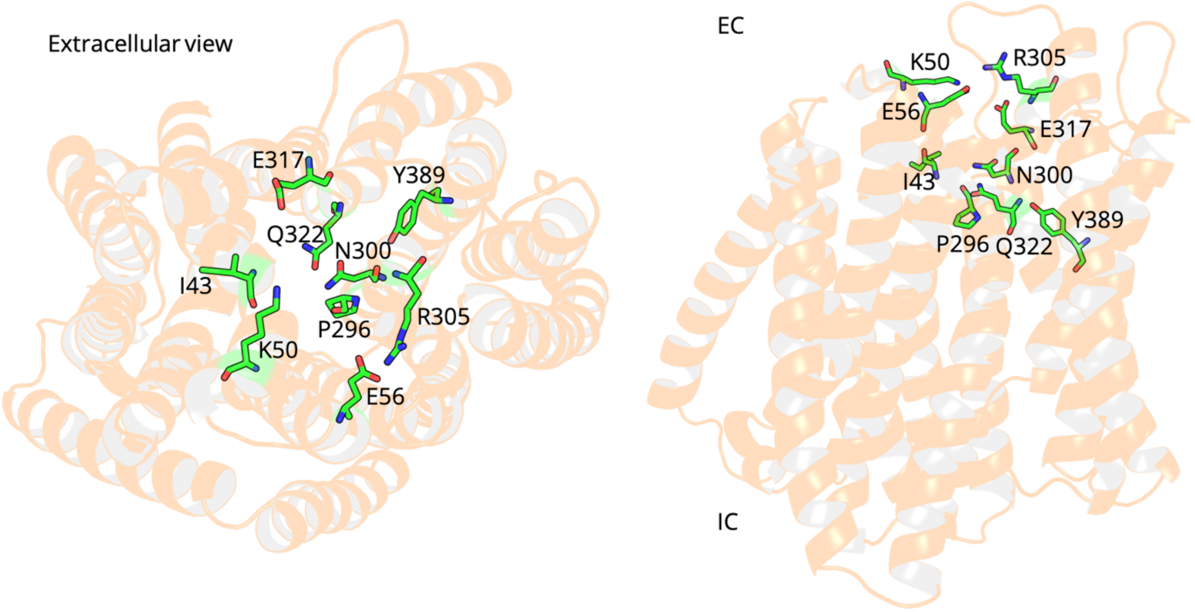

Supporting Fig. S8: Interactions on the extracellular side in Occluded conformation

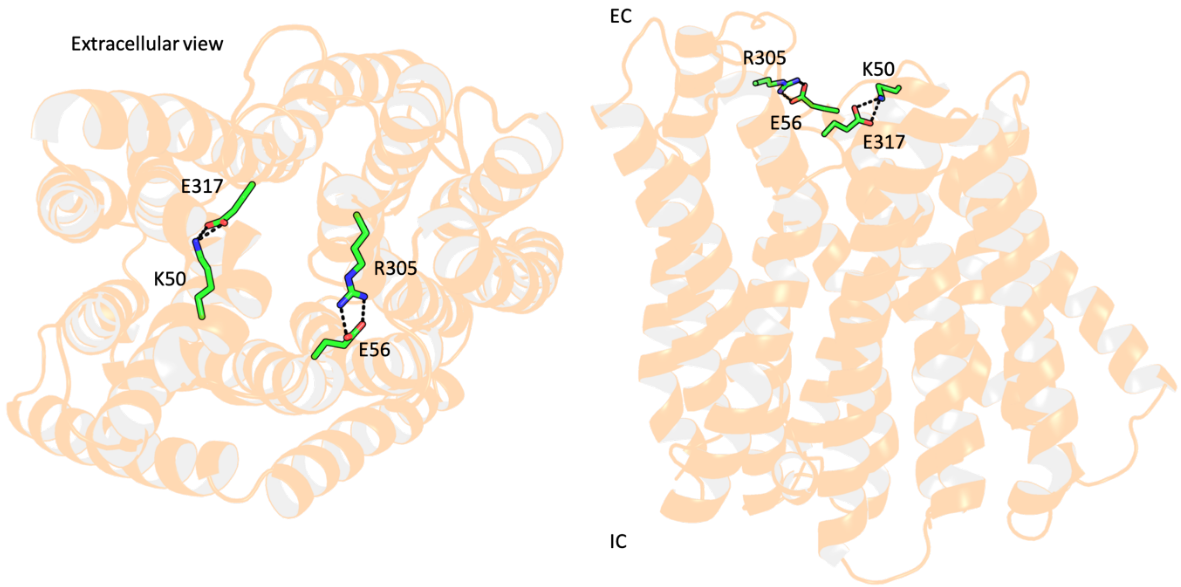

Supporting Fig. S9: E56-R305 and K50-E317 ion pair interactions in the Occluded conformation.

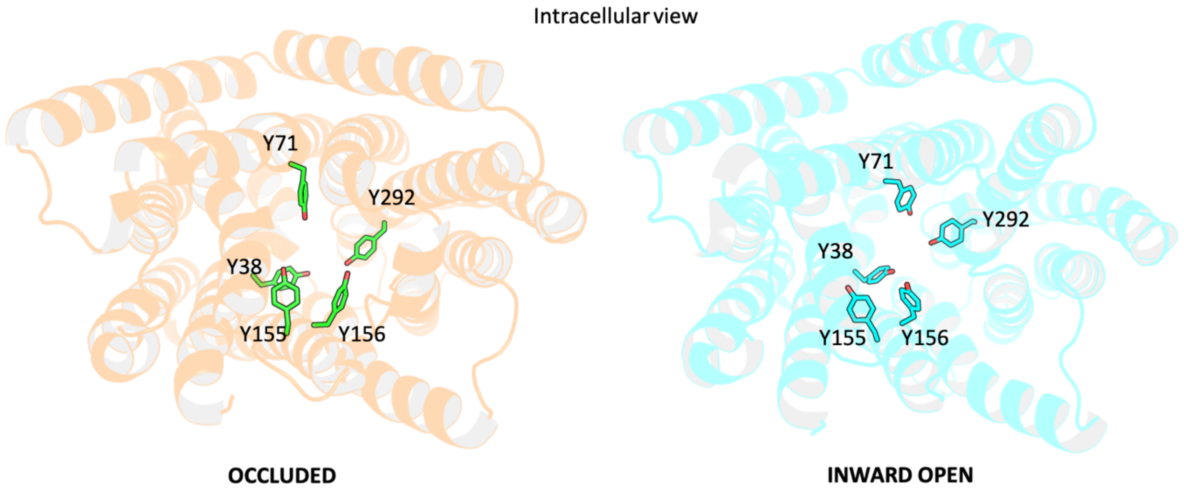

Supporting Fig. S10: The rotation of Tyrosine residues in the Occluded and Inward Open conformations

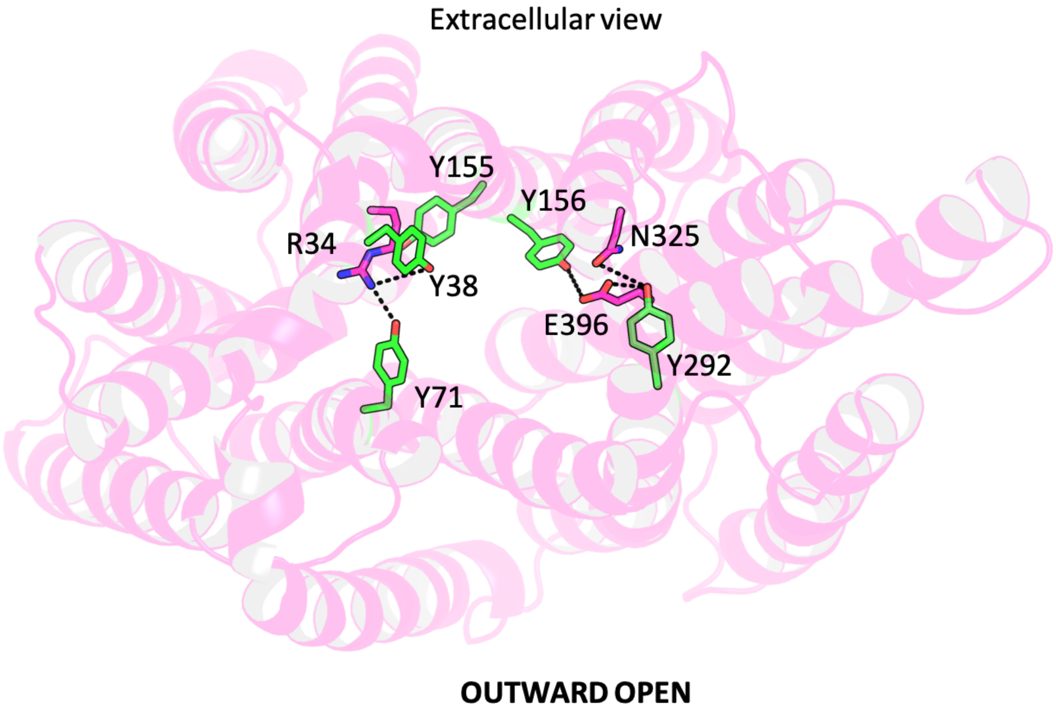

Supporting Fig. S11: The conformation of the tyrosine residues in the Outward Open conformation

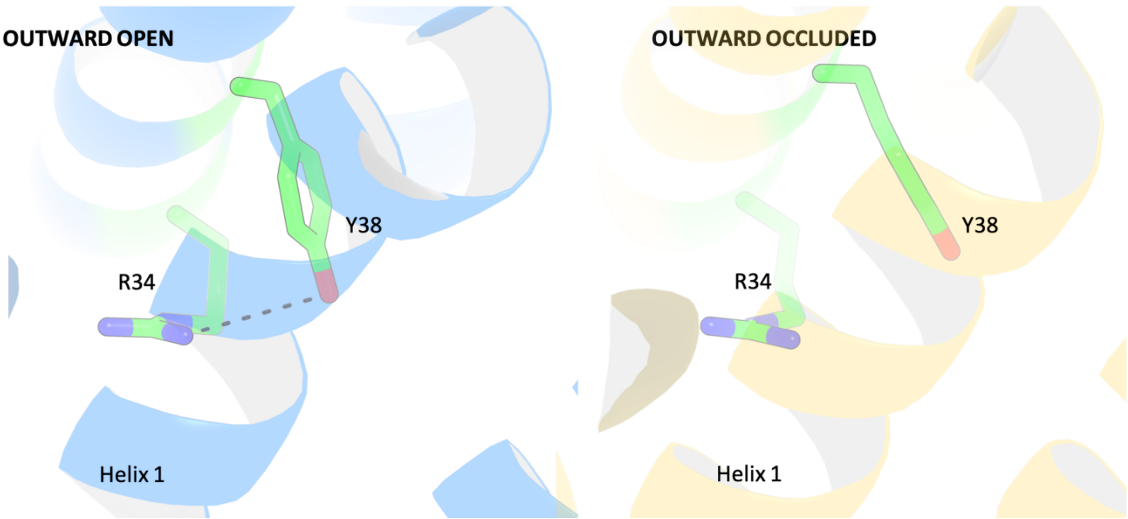

Supporting Fig. S12: Hydrogen bonding interactions between R34 and Y38 in the Outward Open (blue) and Outward Occluded (yellow) states.

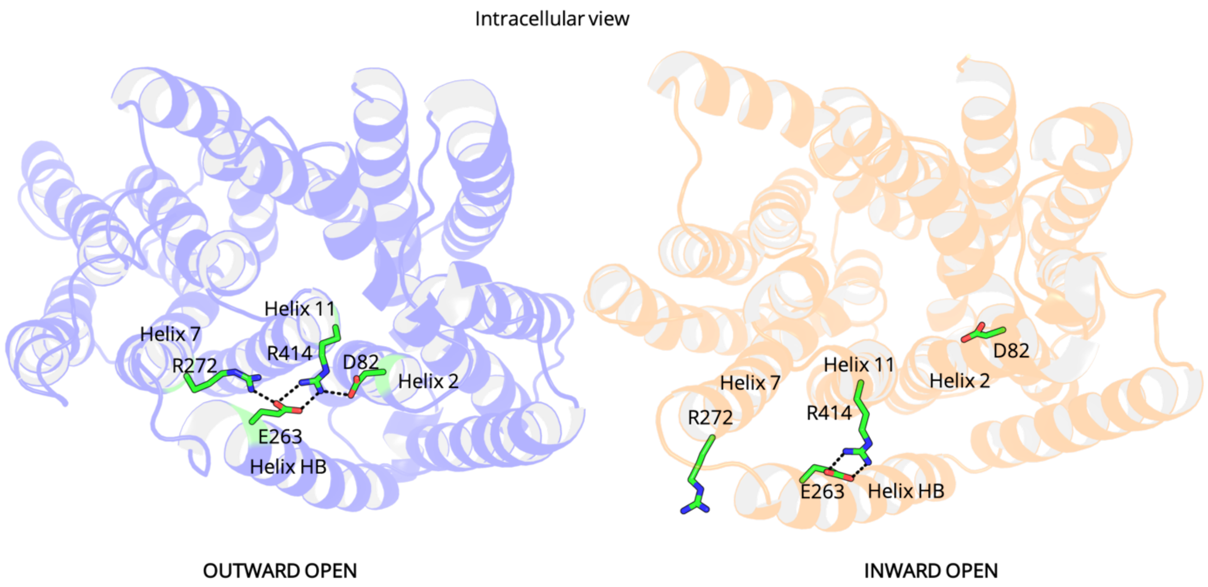

Supporting Fig. S13: The interactions in Cluster 1 in the Outward Open (blue) and Inward Open (orange) conformations

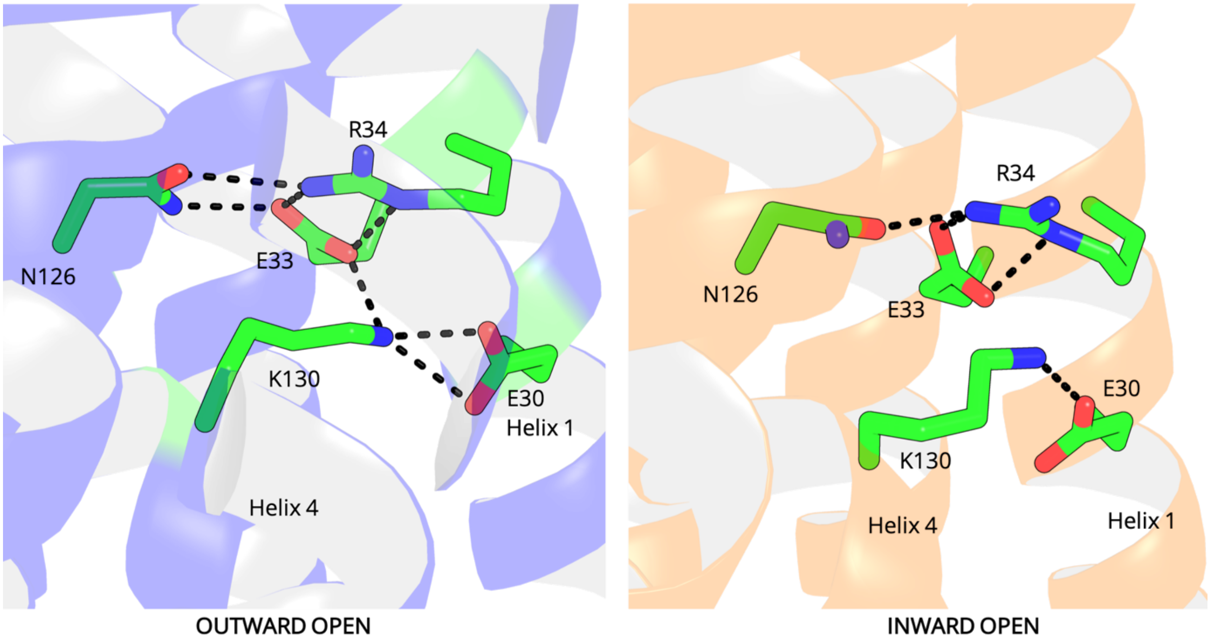

Supporting Fig. S14: The interactions in Cluster 2 in the Outward Open (blue) and Inward Open (orange) conformations

References

[1] Bateman, A., Martin, M. J., Orchard, S., Magrane, M., Ahmad, S., Alpi, E., Bowler-Barnett, E. H., Britto, R., Bye-A-Jee, H., Cukura, A., Denny, P., Dogan, T., Ebenezer, T. G., Fan, J., Garmiri, P., da Costa Gonzales, L. J., Hatton-Ellis, E., Hussein, A., Ignatchenko, A., … Zhang, J. (2023). UniProt: the Universal Protein Knowledgebase in 2023. *Nucleic Acids Research*, *51*(D1), D523–D531. https://doi.org/10.1093/nar/gkac1052

[2] Berman, H. M., Westbrook, J., Feng, Z., Gilliland, G., Bhat, T. N., Weissig, H., Shindyalov, I. N., & Bourne, P. E. (2000). The Protein Data Bank. In *Nucleic Acids Research* (Vol. 28, Issue 1). http://www.rcsb.org/pdb/status.html

[3] Guettou, F., Quistgaard, E. M., Trésaugues, L., Moberg, P., Jegerschöld, C., Zhu, L., Jong, A. J. O., Nordlund, P., & Löw, C. (2013). Structural insights into substrate recognition in proton-dependent oligopeptide transporters. *EMBO Reports*, *14*(9), 804–810. https://doi.org/10.1038/embor.2013.107

[4] Jumper, J., Evans, R., Pritzel, A., Green, T., Figurnov, M., Ronneberger, O., Tunyasuvunakool, K., Bates, R., Žídek, A., Potapenko, A., Bridgland, A., Meyer, C., Kohl, S. A. A., Ballard, A. J., Cowie, A., Romera-Paredes, B., Nikolov, S., Jain, R., Adler, J., … Hassabis, D. (2021). Highly accurate protein structure prediction with AlphaFold. *Nature*, *596*(7873), 583–589. https://doi.org/10.1038/s41586-021-03819-2

[5] Paysan-Lafosse, T., Blum, M., Chuguransky, S., Grego, T., Pinto, B. L., Salazar, G. A., Bileschi, M. L., Bork, P., Bridge, A., Colwell, L., Gough, J., Haft, D. H., Letunić, I., Marchler-Bauer, A., Mi, H., Natale, D. A., Orengo, C. A., Pandurangan, A. P., Rivoire, C., … Bateman, A. (2023). InterPro in 2022. *Nucleic Acids Research*, *51*(D1), D418–D427. https://doi.org/10.1093/nar/gkac993

[6] Pravda, L., Sehnal, D., Toušek, D., Navrátilová, V., Bazgier, V., Berka, K., Vařeková, R. S., Koča, J., & Otyepka, M. (2018). MOLEonline: A web-based tool for analyzing channels, tunnels and pores (2018 update). *Nucleic Acids Research*, *46*(W1), W368–W373. https://doi.org/10.1093/nar/gky309

[7] Wahler, D., Schauser, L., Bendiek, J., & Grohmann, L. (2013). Next-Generation Sequencing as a Tool for Detailed Molecular Characterisation of Genomic Insertions and Flanking Regions in Genetically Modified Plants: A Pilot Study Using a Rice Event Unauthorised in the EU. *Food Analytical Methods*, *6*(6), 1718–1727. https://doi.org/10.1007/s12161-013-9673-x

[8] Webb, B., & Sali, A. (2016). Comparative protein structure modeling using MODELLER. *Current Protocols in Bioinformatics*, *2016*, 5.6.1-5.6.37. https://doi.org/10.1002/cpbi.3

[9] Zhang, Y., & Skolnick, J. (2005). TM-align: A protein structure alignment algorithm based on the TM-score. *Nucleic Acids Research*, *33*(7), 2302–2309. https://doi.org/10.1093/nar/gki524
